# Spices, Condiments, Extra Virgin Olive Oil and Aromas as Not Only Flavorings, but Precious Allies for Our Wellbeing

**DOI:** 10.3390/antiox10060868

**Published:** 2021-05-28

**Authors:** Irene Dini, Sonia Laneri

**Affiliations:** Department of Pharmacy, University of Naples Federico II, Via Domenico Montesano 49, 80131 Naples, Italy; slaneri@unina.it

**Keywords:** spices, condiments, extra-virgin olive oil, antiviral properties, antioxidant properties, nutricosmetic

## Abstract

Spices, condiments and extra virgin olive oil (EVOO) are crucial components of human history and nutrition. They are substances added to foods to improve flavor and taste. Many of them are used not only to flavor foods, but also in traditional medicine and cosmetics. They have antioxidant, antiviral, antibiotic, anticoagulant and antiinflammatory properties and exciting potential for preventing chronic degenerative diseases such as cardiomyopathy and cancer when used in the daily diet. Research and development in this particular field are deeply rooted as the consumer inclination towards natural products is significant. It is essential to let consumers know the beneficial effects of the daily consumption of spices, condiments and extra virgin olive oil so that they can choose them based on effects proven by scientific works and not by the mere illusion that plant products are suitable only because they are natural and not chemicals. The study begins with the definition of spices, condiments and extra virgin olive oil. It continues by describing the pathologies that can be prevented with a spicy diet and it concludes by considering the molecules responsible for the beneficial effects on human health (phytochemical) and their eventual transformation when cooked.

## 1. Introduction

Spices and condiments have played an essential role in human nutrition and participated in developing most cultures worldwide. The use of curry was known in 2000 B.C.E. in India. In Egypt and Babylon, spices such as garlic, cumin and coriander were considered magical. The Greeks and Romans used anise, savory, basil, garlic, hyssop, fennel, mustard, capers, cumin, coriander, oregano, myrtle, parsley, verbena in the kitchen, medicine and cosmetics. Marco Polo in the 13th and the European colonization of Africa, America and Asia during the 15th to 17th centuries improved and spread condiments and spices worldwide [1]. Spices and cooking processes contribute to the ethnic identity of food [2]. Ethnic foods have increased their popularity among consumers worldwide since tourism, international trade and immigration raised the possibility of tasting them. Social media and the opportunity to share culinary experiences also contributed [3,4,5,6]. Partly driven by the improved popularity of ethnic food consumption, the global seasoning and spices market was USD 136.24 billion in 2019 and its growth rate is probable to grow by 4.8% from 2015 to 2025 steadily. The global seasoning and spices market size was valued at USD 13.77 billion in 2019 and is expected to grow at a compound annual growth rate (CAGR) of 6.3% from 2020 to 2027 [7]. The nutrients and phytochemicals in spices, extra virgin olive oil and flavorings are widely used in traditional medicine, pharmaceuticals, dental preparation, aromatherapy and nutraceuticals [8]. The Dietary Supplement and the Education Act have defined “nutraceuticals” as supplements containing herbs, plant products, metabolites, or extracts singly or combined [9]. Currently, the cosmetics industry uses spices and extra virgin olive oil to prepare food supplements and topical skincare cosmetics to combat blemishes from the inside and outside simultaneously [10,11,12,13]. In this study, the nutraceutical potential of spices, herbs and condiments, is revised to demonstrate the fundamental role of safeguarding our health that they have in the diet. Special attention is paid to the transformations that can occur during cooking, the synergistic effects linked to the simultaneous use of more than one seasoning and any toxic effects to maximize the biological impact and avoid any side effects. A brief mention of extra virgin olive oil properties is given since it contributes synergistically with spices, herbs and condiments to the nutraceutical value of the finished dish when used to flavor foods.

## 2. What Differentiates Spices from Herbs, Condiments, Aromas and Extra Virgin Olive Oil?

According to the Codex Alimentarius, “herbs, spices, seasonings and condiments” are considered food flavoring substances usually from botanical sources, dehydrated, ground or whole, added food to improve aroma and taste. Instead, extra virgin olive oil is contained in the section “fat and oils” [14]. Spices, herbs, salt, salt substitutes, vinegar, seasonings, condiments, mustards, sauces, soups and broths, salads, spreads, soy-based condiments, protein products from sources other than soy, yeast and similar products are a part of “herbs, spices, seasonings, and condiments” section.

### 2.1. Spices and Herbs

Spices are mixtures in powder or paste form, such as chili seasoning and curry paste. They are sometimes dried before use. Spices are obtained from the bark, bulbs (e.g., onion and garlic), fruits (e.g., peppers and star anise), flowers (orange and lavender, seeds (e.g., fennel, coriander, sesame and cumin), roots (e.g., ginger and turmeric), or the entire plant (e.g., cinnamon) [15]. Spice plants are often used as sources of phytochemicals and essential oils (Eos) [16]. Phytochemicals are bioactive substances which quality and content in spices depend on plant variety, part of the plant, pedoclimatic condition, harvest period, drying, type of processing and storage [15]. The Eos in spices are aromatic oily liquids containing pharmacologically active components (principally terpenoid and phenolic compounds). Eos are obtained by steam distillation, cold-soaking, extrusion, or solvent extraction [17].

The “herbs or culinary herbs” are plants with aromatic leaves, stems and flowers such as basil, parsley, rosemary and oregano.

### 2.2. Condiments and Seasonings

Condiments are prepared food flavoring containing spices or spices extractives in single forms (e.g., onion salt, garlic salt) or mixtures of constituents (e.g., mustard, chili sauce) which are added to food during cooking and/or eating [18]. They are available in liquid, semisolid and solid forms. The food category “condiments”, in the Codex Alimentarius, does not contain condiment sauces (e.g., mayonnaise, ketchup, mustard) or relishes. Sauces are liquid or semi-liquid products able to enhance the appearance, aroma and flavor of foods. They may or not include spice or spice extracts [18]. Seasoning is the method of adding salts, spices, or herbs to food to improve the flavor. In the last years, condiments, seasonings and spices are used as vehicles for micronutrient fortification since they are cheap and widely consumed by people of all socioeconomic backgrounds [19]. Their colors and flavors mask undesirable organoleptic characteristics from fortification and intensely flavored condiments avoid the overconsumption of a fortified nutrient. Soy sauce, bouillon cubes and fish sauce are used as vehicles to enhance iron consumption [20]. Bouillon cubes are dry broth made by dehydrated meat stock or vegetables. In West Africa, where the bouillon industry is more enhanced than the salt production industry, bouillon cubes are fortified with iodized salt alone or combined with zinc, iron, folic acid, vitamin A, or other B vitamins [21,22].

## 3. Condiment

### 3.1. Vinegar

Vinegar is a common condiment worldwide. It is obtained from the transformation of sugars to ethanol by yeasts and ethanol’s oxidation to acetic acid by bacteria in cider, wine, malt, grain, spirit, raisin and other fruit [23]. It is possible to buy a spray-dried vinegar powder, which provides maximum vinegar taste and may be rehydrated with water in a ratio of 1:1/vinegar:water. The health benefits of vinegar are due to acetic acid and other organic acids (mainly acetic acid and lactic acid together with malic acid, tartaric acid, citric acid and succinic acid), amino acids, phenolic compounds (e.g., catechin, chlorogenic acid, syringic acid, ferulic acid, protocatechuic acid, caffeic acid, gallic acid and *p*-coumaric acid), flavanols (e.g., epicatechin), flavonols (e.g., rutin), anthocyanidin (e.g., malvidin-3-glucoside), anthocyanin (e.g., pyranoanthocyanin), carotenoids, vitamins (B group and C), minerals (Al, Ca, Cr, Fe, K, Mn, Mg, Na, P, S and Zn), alkaloids and sugars (glucose, arabinose, fructose, xylose and mannitose) able to induce antioxidant, antitumor, antidiabetic, antihypertensive, antiobesity, antiinflammatory and antimicrobial effects [24,25]. The bioactive compounds’ composition and concentration depend on the raw material used to produce the vinegar and the production method [26]. Cosmetic products based on vinegar are formulated to counteract the signs of skin aging [27].

### 3.2. Extra-Virgin Olive Oil

**Figure 1:**
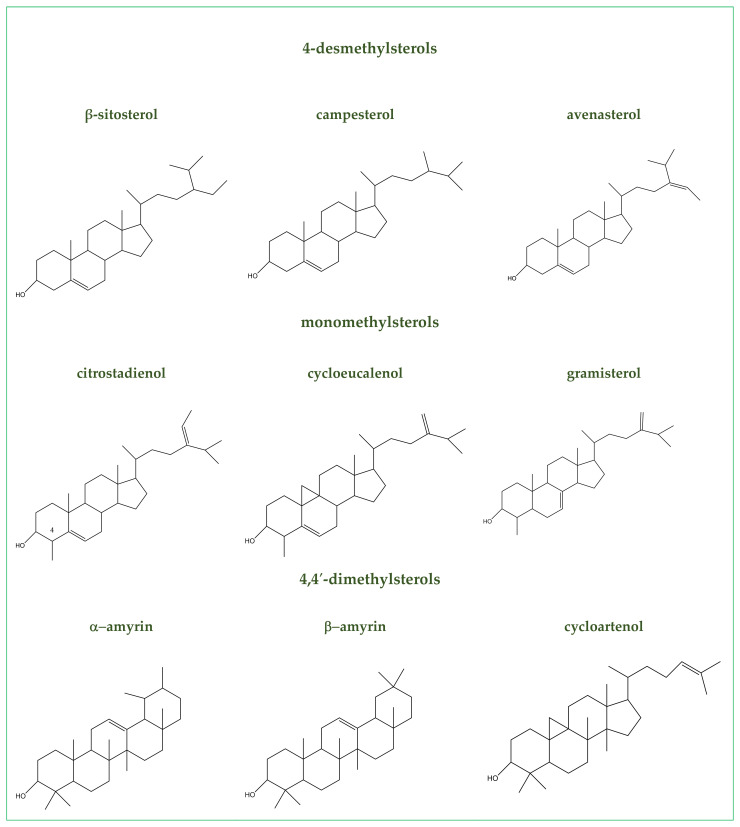
Representative sterol’s identified in EVOO.

Extra virgin olive oil (EVOO) is described in Section 2 of the Codex Alimentarius: “Standards for Fats and Oils from Vegetable Sources”. EVOO is made from the olive tree’s fruit by mechanical processes and purified by washing with water, filtering, settling and centrifuging only [28]. It is often debated whether EVOO should be considered a condiment or a functional condiment since it is used to enhance the appearance, aroma and flavor of foods and it is rich in nutrients and natural antioxidants like a functional food. The healthy and nutritional values are ascribable to monounsaturated fatty acids (MUFAs: 16:1; 18:1; 20:1), polyunsaturated fatty acids (PUFAs), squalene, triterpenic acids, phytosterols, dialcohols, polyphenols and tocopherols [29,30]. Oleic acid (55.0–83.0% of the lipid content), palmitic (7.5–20.0% of the lipid content), linoleic (3.5–21.0% of the lipid content), stearic (0.5–5.0% of the lipid content), palmitoleic (0.3–3.5% of the lipid content), linolenic acids are the more representative fatty acids in the EVOO. Instead, myristic, eicosanoic acids, heptadecanoic are present in traces [31]. Olive variety, agronomic conditions and the olives’ ripening affect the fatty acid composition and content. The International Olive Council [32], Codex Alimentarius [33] and European regulations [34,35,36] norm their content in the EVOO. Sterols (or phytosterols) are another group of naturally occurring lipids in EVOO. Their range varies between 800 and 2600 mg/Kg [37]. Three sterols’ classes are identified in the EVOO according to the presence of methylic groups in position C4 in rings A. 4-desmethylsterols are sterols without methyl group (e.g., β-sitosterol, Δ5-campesterol and avenasterol), 4-monomethylsterols have one methyl group (e.g., citrostadienol, cycloeucalenol, obtusifoliol and gramisterol) and 4,4′-dimethylsterols have two methyl groups (e.g., α-amyrin, cycloartenol and β-amyrin) (Figure 1). The most abundant sterol is β-sitosterol (75–90%). The sterol’ levels diminish during the oil’s storage and enhance peroxides [38]. The most concentrated phenolic compounds in the EVOO are lignans (e.g., pinoresinol, hydroxypinoresinol, acetoxypinoresinol), followed by secoiridoids (e.g., ligstroside, ligstroside decarboxymethyl aglycone, oleuropein, oleuropein aglycone mono-aldehyde, etc.), phenolic alcohols (e.g., tyrosol), flavones (e.g., luteolin, apigenin), flavonols (e.g., quercetin-3-rutinoside), anthocyanidin, (e.g., cyanidin glucosides) and phenolic acids (e.g., vanillic acid, ferulic acid, cinnamic acid, hydroxybenzoic acid) [39]. These substances modulate aging-associated processes and have antiviral, antitumor, anti-atherogenic, antihepatotoxic, antiinflammatory, immunomodulatory, anti-autoimmune (i.e., rheumatoid arthritis) and hypoglycemic properties [40]. The phenolic compounds profile and concentration in EVOO vary significantly according to the olive cultivar, pedoclimatic factors (altitude and amount of irrigation), agricultural practices [41], oil extraction methods and storage conditions. The EVOO quality is linked to olive fruits free of damage and the absence of pesticide residues (e.g., fungicides, insecticides and herbicides). Biological control using *Trichoderma* species or their metabolites are new options to select the EVOO phenolic profile [40] and terpenoid profiles [42]. The nutraceutical importance of phenolics forced researchers to develop reliable analytical methods for their oil dosage [43]. Moreover, EVOO contains tocopherols [31]. They act as free radical scavengers in membranes and lipoproteins and transform fatty acid peroxyl radicals into tocopheroxyl radicals. α-tocopherol regulates signal transduction, apoptosis pathways and transcriptional regulation of the cell cycle [44].

## 4. Sauces

### 4.1. Soy Products Fermented

Soybeans contain some bioactive compounds such as proteins (e.g., β-conglycinin and glycinin), polyunsaturated fatty acids, lecithin, vitamin E, saponin and isoflavones (e.g., genistein, daidzin, daidzein and glycitein) [45,46]. The fermentation processing method enhances the organoleptic and nutritional properties of soybean products. In fermented soybean products, the isoflavone profiles change (isoflavones aglycone-form increase) and improve the phenols’ total content, flavonoids [47] and bioactive peptides. Bioactive peptides have shown hypotriglyceridemic, hypocholesterolemic, antiobesity, antidiabetic, hypotensive, anticancer, antioxidant and antiinflammatory in experimental models [48].

#### 4.1.1. Soy Sauce

Soy sauce is a liquid seasoning currently, used in cooking worldwide. Studies suggest that soy sauce contains polysaccharides, protein, MUFA (e.g., 18:1 fatty acids), PUFA (18:2 and 18:3 fatty acids), minerals (calcium, iron, magnesium, phosphorus, potassium, sodium, zinc, copper, selenium), vitamins (C, B1, B2, B3 and B6) [49], melanoidins, isoflavones, phenolic acids, furan ketones, peptides, organic acids and β-carbolines [50]. The polysaccharides from soy sauce regulate iron and lipid absorption in the gastrointestinal tract [51]. Soy sauce exhibits antioxidant, antihyperuricemic, antimicrobial, anticarcinogenic, anticataract and antiplatelet activities [52]. Sometimes medicinal herbs are added in soy sauce to improve the antioxidant profile, e.g., citrus peels rich in flavanons (e.g., rutin and hesperidin), flavones (nobiletin), with anticancer, antioxidation, anti-inflammation and cardiovascular diseases protective properties [52].

#### 4.1.2. Miso

Miso is a seasoning made by fermenting soybeans with kōji (the fungus *Aspergillus oryzae*), salt and sometimes barley, rice, seaweed or other ingredients. It contains vitamins (B2, B12, E), fat, protein, minerals (iron, calcium, sodium), carbohydrates, [53], saponin, lecithin, phenolic acids (ferulic, vanillic, p-OH-benzoic, p-coumaric, syringic) and isoflavonoids (daidzein, genistein) [54,55]. Miso has antioxidant, ACE-inhibitor, stroke preventive, antihypertensive and anticancer properties [56]. Miso improves the absorption of Coenzyme Q10 (CoQ10) supplements. The CoQ10 is a lipid-soluble antioxidant involved in energy production which can improve the symptoms of some geriatric disorders (e.g., glucose metabolism in diabetes, high blood pressure and the symptoms of Parkinson’s disease), decrease peripheral oxidative stress and inflammation [57].

### 4.2. Fish Sauce

Fish sauce is a fermented condiment with a mild fishy flavor, traditionally used in East and Southeast Asian countries [58]. It is obtained by transforming lipids and proteins with enzymes and halophilic microorganisms [59]. Endogenous proteases and proteases produced by microorganisms hydrolyze the proteins in fish into peptides and amino acids [60,61]. The amino acids in fish sauces contribute to the umami taste and have some biological activity among these anti-oxidative, antithrombotic, hypocholesterolemic, antidiabetic and antihypertensive effects are reported [62,63]. Moreover, they inhibit the ACE enzyme (angiotensin I-converting enzyme) [64], able to stabilize blood pressure by transforming angiotensin I to the potent vasoconstrictor angiotensin II and inactivating the vasodilator peptide bradykinin [65]. The fish sauce contains vitamins (A, B1, B2, B3 and B9), fat, protein, minerals (iron, calcium and phosphorus), carbohydrates [66,67] and high levels of docosahexaenoic acid [68] are able to regulate the symptoms of atopic dermatitis [69].

### 4.3. Tabasco

Tabasco is red pepper hot-sauce sauce obtained by lactic acid fermentation due to autochthonous bacteria [70]. Sodium chloride is added to the peppers to make the pulp microbiologically safe. It selects homofermentative lactic acid bacteria and destroys enterobacteria [71]. After four weeks, vinegar and salt are added to pepper extract to obtain tabasco sauce. Tabasco contains vitamins (C, B1, B2, B3, B5, B6, B9, A and E), fat, protein, minerals (iron, calcium, magnesium, potassium, sodium and phosphorus), carbohydrates [72]. Enzymatic hydrolysis disrupts the cell walls cutting the polysaccharide chains, improve the extract yield and make available bioactive molecules, such as capsaicinoids (capsaicin, dihydrocapsaicin and nordihydrocapsaicin), carotenoids, flavor compounds and polyphenols [73].

## 5. Spices Commonly Used in Food Preparation

### 5.1. Curry

Curry is a combination of spices (turmeric, cumin, coriander, paprika, cardamom and other spices) and herbs and its composition varies between regions [74]. It contains fat, protein, minerals (e.g., iron, calcium, sodium), carbohydrates, fiber [75] and phytochemicals such as flavanols (e.g., catechin), flavonols (e.g., quercetin, kaempferol) [76], carbazole (murrayanol, murrayagetin, marmesin-1”-O-rutinoside, mukoenine-A, -B and C, murrastifoline–F, bis–2-hydroxy-3-methyl carbazole, bismahanine, biskoeniquinone-A and bismurrayaquinone A, koenoline, mukoline, mukolidine). Phytochemicals in curry have antioxidant, antidiabetic, cytotoxic, anticancer, immunomodulatory, antiobesity, antihyperlipidemic, hepatoprotective [77] and skincare activities [78].

### 5.2. Tumeric

Turmeric (*Curcuma longa*) is considered the golden spice of India. It is obtained from the rhizome of a herbaceous plant that belongs to the ginger family *Zingiberaceae*. [79]. Tumeric is widely used as a spice, coloring material, food and preservative in South East Asia, Africa and Brazil. The bright yellow spice is obtained by boiling and drying rhizomes. Turmeric spice has a hot, bitter flavor and a minor fragrance of ginger and orange. It is used to make a curry spice and mustard [80]. The rhizomes contain vitamin C, minerals (e.g., iron, calcium and sodium) [81], flavanols (e.g., catechin), flavonols (e.g., kaempferol and myricetin) [76], curcuminoids (e.g., curcumin, 5-methoxycurcumin, demethoxycurcumin, bis-demthoxycurcumin, cyclocurcumin and dihydrocurcumin), sesquiterpenes (e.g., germacrone, ar-, α, β-turmerones, turmerone, β-bisabolene, zingiberene, α-curcumene, bisacurone, β-sesquiphellandene, curcumenone, procurcumadiol dehydrocurdinone, bisacumol, isoprocurcumenol, curcumenol, epiprocurecumenol, curlone zedoaronediol and turmeronols A and B), steroids (e.g., β-sitosterol, stigmasterol, cholesterol, 2-hydroxymethyl anthraquinone and anthraquinone) and essential oils (e.g., α-phellandrene, cineol, sabinene, sesquiterpenes with turmerones skeleton and borneol) [82]. Tumeric rhizomes are used as stimulants, stomachs and blood purifiers to prevent anorexia, diabetic wounds, hepatic disorders, rheumatism, sinusitis, bronchitis, asthma, skin infections and eye infections [82].

### 5.3. Fenugreek

Fenugreek (*Trigonella foenum-graecum* Linn.) belongs to the *Fabaceae* family. The leaves, seeds and flowers are used dry. The seeds release a maple–curry–nutty flavor by crashing. Leaves and sprouts have a sweeter taste than the seeds and are eaten as a vegetable and mixed into dough, stews and beans. It contains amino acids (glutamic acid, aspartic acid, leucine, tyrosine, phenyl cysteine and alanine), fatty acids (e.g., mono- and di-galactodiacylglycerols, oleic acid, linolenic acid, linoleic acid, glycolipids, phosphatidylethanolamine and phosphatidylcholine), vitamins (e.g., A, B1, B2, C, niacin, nicotinic acid and folic acid) and minerals (e.g., Fe, P, Ca, Mg, S, Cu, Co, Zn, Mn and Br) [83]. The phytochemical analysis of fenugreek has revealed the presence of furostanols (e.g., protodioscin derivatives) and spirostanols (e.g., dioscin derivatives) saponins, steroids, alkaloids (e.g., trigonelline), flavonols (e.g., quercetin-3-O-rhamnoside), flavons (e.g., vitexin-7-O-glucoside, apigenin-6-C-glucoside, apigenin-6-C-glucoside, apigenin-8-C-glucoside, apigenin-6-C-xyloside-8-C-glucoside, apigenin-6 and 8-C-diglucoside), isoflavonoids (e.g., maackiaian and medicarpin), terpenes and phenolic acid derivatives (e.g., caffeic acid, p-coumaric acid and chlorogenic acid, hymecromone, trigocoumarin, trigoforin, scopoletin and γ-schizandrin) [83]. Pharmaceutical employment of fenugreek is related to diabetes, obesity, hyperlipidemia, inflammation damages, cancer, oxidative stress reparations and improving women’s health [83].

### 5.4. Garlic

Garlic (*Allium sativum*) is an herb of the Liliaceae family. *Allium* is derived from the Celtic word al (burning, pungent). The bulb is widely used as a culinary spice and in traditional medicine [84]. It contains vitamins (e.g., A and C) [85] and some bioactive compounds such as flavanols (e.g., catechin), flavonols (e.g., kaempferol, myricetin and quercetin) [76], organosulfur compounds (e.g., allicin, diallyl sulfide, diallyl disulfide, diallyl trisulfide, S-allyl-cysteine, E/Z-ajoene and alliin), phenolic compounds (e.g., β-resorcylic acid, pyrogallol, gallic acid and protocatechuic acid), saponins (e.g., proto-desgalactotigonin, desgalactotigonin-rhamnose, proto-desgalactotigonin-rhamnose, sativoside B1-rhamnose, voghieroside D1 and sativoside R1) and polysaccharides [86,87,88,89,90]. Garlic has antioxidant, antiinflammatory, antiobesity, antidiabetic, anticancer, cardiovascular protective, immunomodulatory and antibacterial properties [91]. The antioxidant properties of garlic are related to organosulfur compounds, flavonoids and saponins. Garlic improves and regulates the antioxidant enzyme activities (heme oxygenase-1 and the glutamate-cysteine ligase modifier) and the nuclear erythroid 2-related factor 2 (Nrf2-ARE) pathway [92,93]. Garlic could constrain inflammation by impeding inflammatory mediators’ action (e.g., nitric oxide, tumor necrosis factor-α and interleukine-1). It decreases nitric oxide production and prostaglandin E-2 by reducing the expression of inducible NO synthase, cyclooxygenase-2 and the transcription of the nuclear factor-kappa B [94,95]. The main immune-modulating components in garlic are polysaccharides. They have an immunomodulatory effect and regulate the expressions of tumor necrosis factor-α, IL-6, IL-10 and interferon-γ in macrophages. Polysaccharides in fresh garlic exhibit a more potent activity on the immune system than fermented garlic since the fructans degrade during processing [96]. Garlic’s cardiovascular protective effects are related to inhibition of oxidative stress and lipid peroxidation, control of angiotensin-converting enzymes and NO and H_2_S production. Moreover, garlic powder can reduce blood pressure, cholesterol (total and low-density lipoprotein cholesterol) and platelet aggregation [91]. It decreases hypertension by reducing oxidative stress, improving NO and hydrogen sulfide production and inhibiting the angiotensin-converting enzyme [92]. Garlic prevents different cancer pathologies by regulating carcinogen metabolism, decreasing cell growth and proliferation, inducing apoptosis, destructing angiogenesis and preventing invasion and migration [91]. Garlic enhances gastrointestinal functions and relieves gastric ulcers and colitis, by decreasing inflammation, oxidative stress and Helicobacter pylori levels [91]. Finally, fermented garlic reduces obesity by impeding lipogenesis and controlling lipid metabolism [91].

### 5.5. Ginger

Ginger (*Zingiber officinale*) rhizome is consumed as a fresh paste, dried powder, slices preserved in syrup, crystallized ginger, or tea flavoring. It contains carbohydrates, protein, free amino acids, fatty acids, triglycerides, ash, crude fiber [97,98,99], minerals (e.g., potassium, copper, magnesium, silicon, manganese), vitamins (e.g., A, E, C, B1, B2, B3, B5, B6, B9 and B12) [100,101], flavanols (e.g., catechin), flavonols (e.g., myricetin) [76], oleoresin (e.g., sesquiterpene hydrocarbons), phenolic compounds (e.g., gingerole, shogoals), diasyleheptanoids (e.g., gingerenone), curcuminoids (e.g., curcumin), alkaloids, carotenoids, tannins, flavonoids, saponins, cardinolides and steroids [100]. Ginger has antioxidant, antiinflammatory, anticancer, hypocholesterolemic, cardio preventive, antibiotic and antimicrobial effects [102].

### 5.6. Chilli Pepper

Chilli pepper (*Capsicum annuum*) is a well-known domesticated species of the genus *Capsicum*. It contains vitamin C, carotenoids (e.g., β-carotene, antheraxanthin, violaxanthin, zeaxanthin, capsanthin, capsorubin and lutein), capsaicinoids, phenolic acids (e.g., chlorogenic acid, caffeic acid, ferulic acid, coumaric acid), flavonols (e.g., rutin and quercetin) and flavanones (e.g., hesperidin), [103,104]. Health-promoting chilly pepper activities are associated with antioxidants and antiinflammatory activities of carotenoids and phenols [104]. It has chemopreventive, antidiabetic, antiobesity, cardioprotective, hepatoprotective and photoprotective skin properties [104].

## 6. Herbs Commonly Used in Food Preparation

### 6.1. Basil

*Ocimum basilicum* L., belonging to the Lamiaceae family, contains polysaccharides, vitamins, minerals (e.g., magnesium, calcium, iron and zinc), fatty acids (e.g., stearic acid, oleic acid, palmitic acid, linoleic acid, myristic acid, α-linolenic acid, capric acid, lauric acid and arachidonic acid), steroids, phenolic acids (e.g., caffeic acid, vanillic acid, rosmarinic acid, chlorogenic acid and p-hydroxybenzoic), flavonols (e.g., quercetin and rutin), flavones (e.g., apigenin) [105], β-carotene, terpenes, alcohols, aldehydes, ketones esters and ethers [106]. Biological effects of basil include antioxidant, anticancer, anti-atherosclerotic, hypolipidemic, antidiabetic, immunity boost and antiaging activities [107].

### 6.2. Parsley

Parsley (*Petroselinum crispum*) belongs to Apiaceae (Umbelliferae) family. It contains flavones (e.g., diosmetin 7-malonylapiosylglucoside, diosmetin/chrysoeriol 7-malonylapiosylglucoside, diosmetin 7-apiosylglucoside, apigenin dihexoside and apigenin 7-apiosylglucoside), flavonols (e.g., isorhamnetin 3-alonylglucoside-7-glucoside, isorhamnetin 3-malonylglucoside-7-glucoside, isorhamnetin 3-glucoside, apigenin 7-malonylapiosylglucoside, apigenin 7-malonylapiosylglucoside, apigenin 7-malonylapiosylglucoside, apigenin 7-dimalonylapiosylglucoside and apigenin 7-glucoside), flavanones (e.g., hesperetin 7-glucoside), carotenoids (e.g., beta-cryptoxanthin, beta-carotene, lutein and zeaxanthin), coumarins (bergapten, psoralen and xanthotoxin), tannins and triterpenes [108]. Biological activities ascribed to parsley are antidiabetic, antihypertensive, antioxidant, antitumorigenic and gastroprotective activities [109].

### 6.3. Fennel

Fennel (*Foeniculum vulgare* Mill.) is a perennial, umbelliferous herb. Stems, leaves and shoots are used in culinary traditions. It contains carbohydrates, essential fatty acids (e.g., ω-6 and ω-3) [110], phenylpropanoids (e.g., estragole and trans-anethole), monoterpenes (e.g., α-pinene and α-phellandrene), hydrocinnamic acids (e.g., neochlorogenic acid, chlorogenic acid, criptochlrogenic acid, 5-feruoylquinic acid,1,4-O-dicaffeoylquinic acid and 1,5-O-dicaffeoylquinic acid) and flavonols (e.g., quercetin-3-O-glucuronide and kaempferol-3-O-glucuronide). Fennel’s beneficial properties include antithrombotic, anticancer, antioxidant, antiinflammatory, hepatoprotective and antidiabetic properties [111].

### 6.4. Sage

Sage (*Salvia officinalis* L.) is an aromatic herb belonging to the Lamiaceae family. It contains carbohydrates, protein, total lipids, MUFA, fiber, minerals (e.g., calcium, iron, magnesium, phosphorus, potassium, zinc, manganese, selenium), vitamins (e.g., C, B1, B2, B3, B6, B9 and E) [112], phenolic acids (e.g., gallic acid, chlorogenic acid, caffeic acid, coumaric acid ferulic acid, rosmarinic acid) flavonols (e.g., quercetin, rutin and myricetin), terpene/terpenoids (camphor, borneol, caryophyllene, elemene, cineole, humulene, ledene, thujone and pinene) [113]. The antioxidant and antiinflammatory properties of sage are related to the terpenoids compounds, phenolic acids and flavonoids [114]. The cryptotanshinone (a diterpenoid) produces an agonistic activity on the opioid system [115]. Terpenes and terpenoids are responsible for anticancer activity. The α-humulene and caryophyllene inhibit breast cancer and colorectal cancer tumor cells; manool induces selective cytotoxicity on human glioblastoma and cervical adenocarcinoma; ursolic acid constrains angiogenesis and invasion of melanoma cells; and rosmarinic acid inhibits the growth of the colon, breast, hepatocellular, prostate, small cell lung carcinomas and chronic myeloid leukemia [114].

## 7. Description of Processes in Which the Phytochemicals and Nutrients in Spices, Condiments and Sauces Can Intervene to Exert Their Beneficial Action

### 7.1. Oxidative Stress

Oxidative stress is produced by an excess of the reactive oxygen species (ROS, e.g., superoxide, hydroxyl radical, hydrogen peroxide and singlet oxygen) and reactive nitrogen species (RNS, e.g., nitric oxide, peroxynitrite, nitrogen dioxide) [116]. ROS production in living organisms is due to phagocytosis, respiratory chain (endogenous reactions), exposure to UV radiation, air pollutants and other physical and chemical agents. The electrons made in the mitochondrial respiratory chain are transferred to molecular oxygen forming superoxide anion. The nitric oxide improves the generation of superoxide anion-producing peroxynitrite enzyme, leading to the increased oxidation of proteins, carbohydrates and lipids. ROS make membrane lipid peroxidation and determines the loss of membrane fluidity, altering cell homeostasis. Humans have endogenous defense mechanisms, such as superoxide dismutase, glutathione peroxidase and catalase, to protect against ROS-induced damage. Improved ROS production alters the balance between oxidant and antioxidant levels, determining a pro-oxidative condition [117]. Oxidative stress is involved in some diseases, including inflammation, atherosclerosis, type 2 diabetes mellitus and cancer [118]. Biomarkers of oxidative stress are lipoproteins oxidation, lipid hydroperoxides, conjugated dienes, malondialdehyde (MDA), F2-isoprostanes (F2-IsoPs), glutathione, protein carbonyls and activities of antioxidant enzymes [119]. A universal index does not identify oxidative stress since the biomarkers used to define the stress status have different kinetics of production and elimination [120]. The assay methods used to control lipid peroxidation determine the levels of lipid peroxides or the end products of lipid peroxidation. [121]. The standard assays to determine protein modifications measure the nitration of protein tyrosine residues and the carbonyl groups of the oxidized proteins [122,123].

### 7.2. The Immune Response

The immune system is a biological system that evolved host protection against viruses, bacteria, fungi, parasites and cancer cells [124]. Four functions of the immune system determine host defense. These include barrier production to stop pathogens, identifying and removing the pathogens that pass the barrier by immune cells and immunological memory creation [125]. Physical barriers are skin, respiratory, gastrointestinal tract (including microbiota), nasopharynx, hair and cilia. Immune cells are granulocytes (neutrophils, basophils and eosinophilic), lymphocytes (T-, B- and natural killer-cells) and phagocytes (monocytes, macrophages, dendritic cells and mast cells) [126]. Cellular and humoral responses can be innate and adaptive in the identification and eradication of pathogens. The innate responses are non-specific responses to pathogens that occur when there is no previous exposure or immunization. This action’s actors are physical barriers, biochemical mechanisms, inflammatory response, complement system and phagocytes [127]. The speed and effectiveness of these responses are independent of the number of exposures to the pathogen. The adaptive responses are linked to the immunological “memory” and can generate an antigen-specific response. They involve antigen-specific T lymphocytes, which determine the adaptive response or destroy virally infected cells and B lymphocytes can secrete immunoglobulins (antibodies specific against the infecting pathogen) [127]. When the adaptive immune responses occur, the T helper cells (Th1 and Th17) migrate into circulation from lymphoid tissue, penetrate infected sites and make cytokines. The innate and adaptive immune responses control inflammation and the progress of the self and non-self-discrimination. Immature T cell populations express antigen-specific receptors that distinguish self or non-self-macromolecules [128]. In the thymus, T lymphocytes with T cell receptors (TCRs) recognize the self-peptides and major histocompatibility complex (MHC) proteins destroy nonself-macromolecules [129]. Autoimmune diseases happen when central and or induced peripheral tolerance do not work. Old age, obesity and diet determine the most severe symptoms of the disease. Aging can cause the thymus involution that decreases the output of naive T lymphocytes (T CD8+ kill cells directly and T CD4+, T helper cells that secrete cytokines) [130,131,132,133], the answer to new antigens and an increase of the inflammatory mediators in the blood (inflammageing) [134]. An excessive inflammatory response determines a loss in acquired immunity [134]. Obesity reduces T lymphocytes, B lymphocytes, natural killer cell activity, the antibody and IFN-γ (Interferon-gamma) production [135,136,137]. Food bioactive molecules and micronutrients can increase immune functions [138]. Fatty acids, amino acids, vitamins and mineral ions produce leukotrienes, prostaglandins, chemokines, immunoglobulins, cytokines and acute-phase proteins [135,139], valid for the immunity response. Carbazoles and tryptophan-enriched proteins determine antiinflammatory action, activating aryl hydrocarbon receptors. Moreover, diet regulates the microbiota to produce short-chain fatty acids that affect immune responses activating the G-protein–coupled receptors and epigenetic mechanisms [140].

## 8. Nutrient with Potential Nutraceutical Effects

### 8.1. Lipids

Lipids are essential energy sources for the human body. Fatty acids are constituents of fats and oils. They are classified into: saturated (without double bond), monounsaturated (with one double bond) and polyunsaturated fatty acids (with some double bond). Polyunsaturated fatty acids (PUFAs) are considered essential acids because humans cannot synthesize them. They are divided into two groups: the omega-3 and omega-6 fatty acids [141]. The free fatty acids (FFAs) serve as energy sources and natural ligands free fatty acid receptors, regulating the secretion of peptide hormones and inflammation. The viruses use the fats to fuse the viral membrane and host cell during replication, endocytosis and exocytosis [142].

#### 8.1.1. Unsaturated Fatty Acids

##### Monounsaturated Fatty Acids’ Health Properties

Monounsaturated fatty acids (MUFAs) are carboxylic acids with hydrocarbon chains having only one double bond. MUFAs inhibit coagulation, improve blood pressure and glucose homeostasis, reduce oxidative states and inflammation and modify plasma lipids, lipoprotein patterns, membrane composition and fluidity of blood cells [29]. In EVOO, the main MUFA by content is the oleic acid (18:1 ω-9), representing 49% to 83% of the total fatty acid. It promotes bile secretion and enhances gastric mucosa protection by decreasing hydrochloric acid secretion [143]. The American Heart Association sets a limit of MUFA consumption at 20% of total energy. The American Diabetes Association and Dietitians of Canada approve almost 25% of energy [144].

##### Polyunsaturated Fatty Acids Health Properties

PUFAs are carboxylic acids with hydrocarbon chains having more than one double bond. PUFAs are classified into ω-3 and ω-6 families. ω-3 fatty (alpha-linolenic, docosahexaenoic and eicosapentaenoic acids) have the double bonds in the third bond from the methyl end. ω-6 acids (linoleic, arachidonic and gamma acids) have it in the sixth bond from the fatty acid’s methyl end [145]. PUFAs are precursors of eicosanoids (mediator signaling molecules). Eicosanoids derived from ω-6 PUFAs are proinflammatory molecules. Eicosanoids from ω-3 PUFAs have anti-inflammatory properties [146]. Arachidonic acid (ARA, n-6), eicosapentaenoic acid (EPA, n-3) and docosahexaenoic acid (DHA, n-3) are lipid immune mediators (SPMs) [147]. Mammals do not synthesize them. Humans transform linoleic acid (LA) and alpha-linolenic acid (ALA) found in foods to n-6 and n-3 long-chain polyunsaturated fatty acids [148]. SPMs contribute to cytokine “kidnapping” and eliminating remnants making the available restoration of structure and tissue homeostasis [149,150,151,152]. They decrease inflammation (duration and magnitude) and accelerate reepithelization, tissue regeneration and wound healing [153]. Moreover, EPA and DHA are the precursors of resolvins and ARA of maresins and protectins. Resolvins, maresins and protectins inactivate polymorphonuclear leukocytes and increase leukocytes, which remove remnants from neutrophil apoptosis (efferocytosis). Finally, EPA and DHA activate nuclear factor kappa B (NFkB), the peroxisome proliferator-activated receptor (PPAR) and destabilize membrane lipid rafts.

### 8.2. Vitamins

#### 8.2.1. Vitamin A

Vitamin A is a fat-soluble retinoid group (retinol, retinyl esters and retinal) [154,155,156]. Retinol is obtained from animal sources as retinyl palmitate, or it can be synthesized in the intestine starting from beta carotene, a precursor/pro vitamin of vegetable origin. Vitamin A controls the differentiation of epithelial tissue, the imprinting of the B and T cells with gut-homing specificity, the arranging T cells and IgA+ cells into intestinal tissues [157], supports the gut barrier [158,159,160], reduces the toxic effects of ROS and regulate the membrane fluidity and gap-junctional communication [161,162]. Vitamin A improves epithelial construction (keratinization, stratification, differentiation) and functional maturation of epithelial cells [163]. It is part of the respiratory and intestine apparatus’s mucus layer, promoting the antigen non-specific immunity function enhancing mucin secretion [163,164,165]. Vitamin A promotes the proliferation and regulation of the thymocytes apoptosis [166,167]. It plays a crucial role in controlling the differentiation, maturation and function of macrophages and neutrophils, which respond to pathogen invasion through phagocytosis and activation of natural killer T cells [168,169]. Vitamin A supervises the early differentiation of the natural killer T (CD4+) and dendritic cells (antigen-presenting cells) [170] and synthesizes immunoglobulins [171]. The balance between T helper 1 and T helper 2 lymphocytes is altered when vitamin A is deficient. Retinoic acid is essential for CD8+ T lymphocyte proliferation and antibody generation by B lymphocytes [172].

#### 8.2.2. B-Group Vitamins

B vitamins are a family of water-soluble vitamins able to act as cofactors and coenzymes in metabolic pathways and play roles in maintaining immune homeostasis [173,174]. B vitamins are obtained from the intestinal microbiota and diet [175]. They contribute to gut barrier function controlling the intestinal immune regulation and are involved in intestinal immune regulation. Vitamin B6 regulates T lymphocyte migration, the folic acid and the T cells in the small intestine [176,177]. Vitamin B12 influences the phagocytic and bacterial killing capacity of the neutrophils [125]. Human gut microbes use vitamin B12 as a cofactor for metabolic pathways [126]. Both vitamins maintain or enhance NK cell cytotoxic activity [177,178,179]. Villarruz-Sulit and Cabaluna (2020) hypothesized that vitamin B supplementation affects the treatment of COVID-19 [179].

#### 8.2.3. Vitamin C

Vitamin C is a water-soluble vitamin. Humans cannot synthesize it since they lack a crucial enzyme in the biosynthetic pathway [180,181]. Vitamin C is an antioxidant vitamin. It can donate electrons [182] and is a cofactor of monooxygenase and dioxygenase (antioxidant enzymes) [183,184]. It is involved in collagen biosynthesis and plays a crucial role in the immune system, including barrier integrity preservation and action on leukocyte function [185,186,187,188,189,190]. It is a cofactor for two enzymes (lysyl and prolyl hydroxylases) needed to maintain the tertiary structure of collagen I [184]. Moreover, vitamin C protects against ROS-induced damage [157], improves keratinocyte differentiation, lipid synthesis [191] and fibroblast proliferation and migration. It acts as an electron donor [192,193,194,195]. Enhances motility/chemotaxis [196,197,198,199,200,201,202,203,204,205], phagocytosis, ROS generation [206,207,208,209,210,211,212,213] and microbial killing [196,197,214,215,216,217]. Moreover, vitamin C facilitates apoptosis, clearance [212,216,218], decreases necrosis and the formation of the extracellular trap (NETosis), the cell death independent of apoptosis [216,218]. Vitamin C improves differentiation and proliferation [204,205,219,220,221,222,223,224,225] of B and T lymphocytes, increases antibody levels [219,224,225,226,227,228], controls the cytokines production [219,229,230,231,232,233,234,235,236,237,238] and decreases the histamine levels [198,204,239,240,241,242,243,244,245].

#### 8.2.4. Vitamin E

Vitamin E is a collection of eight fat-soluble antioxidants that includes α-, β-, γ- and δ-) tocopherol and (α-, β-, γ- and δ-) tocotrienol derivative [246]. Vitamin E determines both humoral and cell-mediated immune functions [247] and enhances infectious pathogens’ susceptibility [248]. It protects membranes from damage caused by free radicals [158,249], regulates NK cell cytotoxic activity [158,179,250,251] and decreases the production of the PGE2 by macrophages [175,252,253]. Vitamin E protects the polyunsaturated fatty acids and immune cells from oxidation [247,254]. It regulates natural killer cell activity, lymphocyte proliferation, specific antibody production following vaccination, the neutrophils’ phagocytosis and promotes interaction between CD4+ T lymphocytes and dendritic cells [125].

### 8.3. Phytochemicals in Spices with Nutraceutical Properties

#### 8.3.1. Phenols

Polyphenols are secondary metabolites of plants, involved in defense against attack by pathogens or UV (ultraviolet) radiation. They are classified based on phenol rings in phenolic acids, flavonoids, stilbenes, curcuminoids and lignans.

##### Flavonoids

The flavonoids consist of two aromatic rings bound together (A and B) and one heterocycle (ring C). They are divided into six subclasses depending on the degree of unsaturation and oxidation of the C ring and the carbon of the C ring on which the B ring is attached: flavones, flavonols, isoflavones, chalcones, anthocyanins, flavanones, flavanols, catechins and proanthocyanidins [255] (Table 1). Flavonoids have antioxidant, anti-mutagenic anti-angiogenic, antibacterial, anti-allergic, antiinflammatory, anticancer, enzyme modulation properties [256,257,258]. They can directly scavenge ROS, stabilize the free radicals, chelate metal ions with phenolic hydroxyl groups, activate phase II detoxification enzymes and block pro-oxidant enzymes. [259]. The flavonoids’ anticancer mechanisms employ the control of the ROS-scavenging enzyme’s activities, autophagy, apoptosis and inhibition of the cancer cell proliferation and invasiveness [259]. The flavonoid’s antiinflammatory actions involve immune cell regulation, suppressing the chemokines, COX-2, cytokines, proinflammatory transcription factors and kappa kinase/c-Jun amino-terminal kinases [260,261]. FDA (Food and Drug Administration) approved a clinical trial of quercetin against Covid-19 [262]. In silico modeling works have shown that quercetin is one of the top five most potent compounds in a database of 8000 small molecules) able to bind the interface site of the ACE2 receptors and theoretically disrupt the initiating infection process of the SARS-CoV-2 Viral Spike Protein [263]. Apigenin, a 5,7-trihydroxyflavon, employs immune-regulatory activity in an organ-specific manner modulating NF-κB activity in the lungs [264], decreasing the secretion of the mast cell [265], T cells [266], COX-2, IL, TNF and NO [267]. In silico modeling, works have shown that apigenin binds the interface site of the ACE2 receptors and has great potential to act as COVID-19 main proteases inhibitors [268]. Isoflavones are phytoestrogens belonging to the non-steroidal estrogens. They improve the adaptive immune system, inhibiting lymphocyte proliferation, antigen-specific immune activities (T- and B-cells) and allergic responses [269,270,271,272]. The isoflavone genistein enhances CD8 T-cells and cytokines’ production by T-cells [269,273,274,275,276]. Phytoestrogens interact with the T-cell and contribute to cytokine responses compartment enhancing or inhibiting the NF-kB pathway. Kojima et al. have shown that they improve gene expression mediated by ROR γ and α (retinoic-acid-receptor-related orphan receptor) in T-lymphoma cells and enhancing the expression of IL-17 [275]. The phytoestrogens interact with the B-cell compartment. The isoflavones inhibit IgG2a (immunoglobulin G2a) antibodies [270,271,272], the antigen-specific IgG1 and IgG3 in thyroiditis [272], the expression of IgE [270], the inflammatory immune response by inhibiting the antigen-presentation and functions of dendritic cells (DCs) [270,271]. They modulate the innate immune system inhibiting the production of IFN-γ, TNF-α, IL-9 and IL-13 from CD4+ T-cells [270,271], suppress allergic inflammation-reducing mast cell degranulation [270,272] and control NK cell activity reducing expression of IL-18Rα (IL-18 receptor α) and IFN-γ production in response to IL-12 and IL-18 [277]. Finally, they induce antiinflammatory responses in macrophages. Dia et al. showed that some phytoestrogens (daidzein and genistein) reduce the production of NO (nitric oxide), the expression of iNOS (inducible nitric oxide synthase) and enhance the superoxide dismutase and catalase activities [278].

##### Curcuminoids

**Figure 2:**
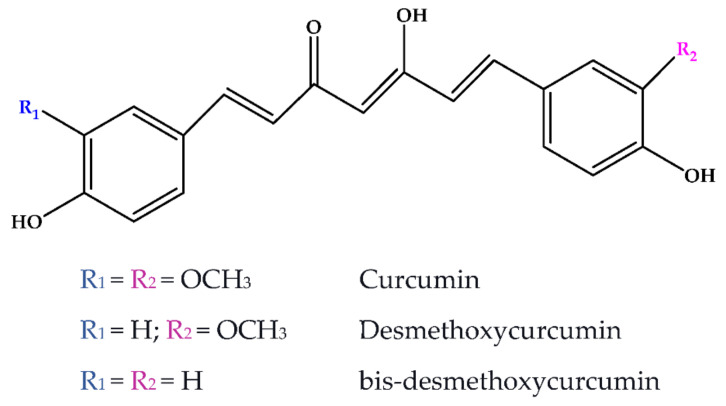
Curcuminoids in turmeric spice.

Curcuminoids are phenolic compounds with neuroprotective, antioxidant, antitumor, anti-acidogenic, antiinflammatory and radioprotective activities [279]. They have a diarylheptanoic nucleus with varying degrees of oxidation and unsaturation (Figure 2).

Curcumin, the principal polyphenol of *Curcuma longa* helps the antiinflammatory system to decrease the metabolism of arachidonic acid, lipoxygenase and cyclooxygenase activities, tumor necrosis factor, interleukins cytokines, nuclear factor-κB and steroids production [280]. It supports antioxidant defense mechanisms, such as scavenge hydroxyl radicals and superoxide anions, protection of cells from DNA damage, lipid peroxidation, protein carbonylation, protein oxidation, improvement of the glutathione’s levels, stabilization of the superoxide-dismutase, glutathione S-transferase and glutathione peroxidase [281] and chelation of the heavy metals (aluminum, cadmium, copper, manganese, zinc and iron) responsible of the ROS production [282]. Curcumin carries out chemopreventive activities improving the glutathione transferase and NADPH quinone reductase (phase 2 detoxification enzymes), reducing cytochrome formation P450 1A1, a pro-carcinogen activating phase 1 enzyme and arachidonic acid production [283]. It has potential as a therapeutic agent for neurological diseases (e.g., Alzheimer’s) since it inhibits amyloid-beta protein aggregation (e.g., α-synuclein, huntingtin, phosphorylated tau, prion proteins, Aβ-oligomers and fibrils) and enhances motor coordination and cognition peroxidase [281]. Finally, curcumin has cardioprotective actions (e.g., antiplatelet and anticoagulant) and improves the activities of detoxifying enzymes (e.g., glutathione-S-transferase) [284].

##### Capsaicinoids

**Figure 3:**
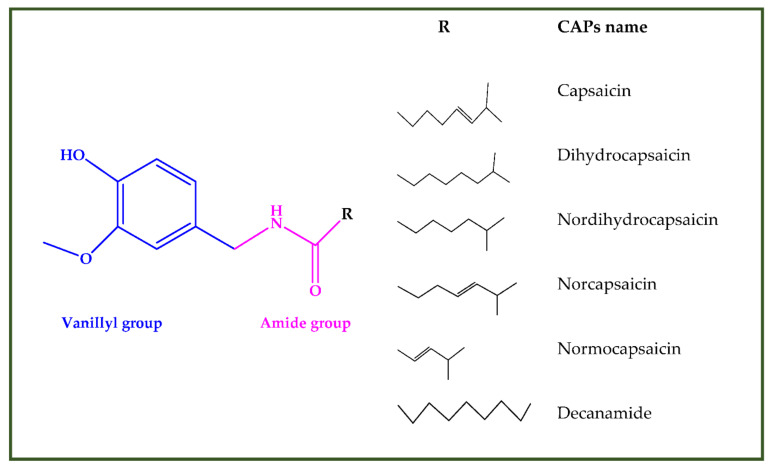
Chemical structures of some capsaicinoids.

Capsaicinoids (CAPs) (Figure 3) are compounds responsible for the burning sensation. Thirteen different CAPs are identified. They are characterized by one vanillyl group, a carboxamide group and a variable aliphatic chain (Figure 3). Capsaicinoids have hypocholesterolemic, antioxidant, antiinflammatory, antitumoral, antidiabetic and antiobesity properties [285]. Capsaicin and dihydrocapsaicin have shown hypocholesterolemic action obtained by reducing cholesterol absorption, improving its hepatic conversion to bile acids, the excretion in the feces and inducing expression of hepatic LDL receptors [286]. Antioxidant activity of CAPs is due to inhibition of lipid peroxidation; radical scavenge formation, depletion of total hepatic thiols and hepatic antioxidant enzyme activities (glutathione-reductase, glutathione-transferase, superoxide dismutase and catalase) [286]. CAPs block the arachidonate metabolites’ production (PgE2, leukotrienes) and the release of the lysosomal enzymes (elastase, hyaluronidase and collagenase) by macrophages [287]. Antitumoral properties are related to the interaction with microsomal xenobiotic-metabolizing enzymes, inactivation of cytochrome P-450 HE1 (and other isoforms of the P-450 family) block of microsomal monooxygenases interested in carcinogen activation [288]. Capsaicinoids have potential application in diabetes prevention since they improve insulin secretion by activating, in islet β-cells, the transient receptor potential vanilloid subfamily member 1 (TRPV1) [289] decrease the concentration of postprandial blood glucose, enhance insulin secretion and glucose tolerance [290]. Finally, capsaicinoids have an antiobesity effect due to their ability to stimulate human brown adipose tissue growth. The human brown adipose tissue is the primary site of non-shivering thermogenesis (NST). It increases whole-body energy expenditure and regulates energy balance and body fatness [291].

#### 8.3.2. Organosulfur Compounds

Organosulfur compounds are biosynthesized for defensive purposes against abiotic stressors by the Allium family’s plants (e.g., garlic and onion). Thiosulfates are transformed by pH, temperature and solvent, into alk(en)yl cysteine sulfoxides, mono- di- and tri-sulfides, S-allyl cysteine, (E)- and (Z)-ajoene and vinyl dithiins [292]. Organosulfur compounds act as anticancer molecules improving the immune system and inducing proliferative signals by converting allyl sulfides into sulfane sulfur [293,294]. They stimulate apoptosis, induce xenobiotic-metabolizing enzymes, enhance the detoxification of carcinogens, have a role in cell cycle arrest and prevent nitrosamines and hydrocarbons’ metabolism, scavenging free radicals and modulate the enzymes responsible for DNA repair [295,296]. Organosulfur compounds have antiinflammatory activities. The allicin inhibits the proinflammatory cytokines from epithelial digestive cells, blocking TNF-α secretion. Diallyl sulfide (DAS), diallyl tri- (DATS), tetra-sulfides and S-allylcysteine (SAC) decreased inflammatory lipopolysaccharide. The allyl methyl disulfide reduces the formation of the IL-8/IP-10 by the TNF-α in intestinal cells [297].

## 9. Effect of Spices and Herbs on the Shelf Life of Foods

In past few years, the protective effects of essential oils (Eos) as antimicrobial and antifungal agents in dairy products (e.g., chicken and meat) have been studied. Essential oils are mixtures of organic chemical compounds from the terpenoid family (mainly mono- and sesquiterpenes), phenols, aldehydes and ketones [298]. Eos have different modes of fungal inactivation: chalcones reduce the synthesis of the cell wall polysaccharide 1,3-beta-D-glucan causing fungal cell wall disruption [299] and decrease the conversion of tubulin into microtubules, causing the interruption of the cell division [300]; aldehydes inhibit fungal cell division by reacting with sulfhydryl involved in fungal cell division and interfere with fungal metabolism forming a charge-transfer complex with electron donors in fungal cells [301]; enones and enals stop fungal growth reacting with nucleophiles in fungi [302]; ascaridole decrease hemin making toxic radicals in the presence of Fe^2+^ [303]; carvacrol causes a breakdown of ion gradients distributing into membranes and interferes with the intracellular calcium homeostasis, improving the passive permeability of the cell membrane, modulating the Ca^2+^ permeable transient receptor channels, preventing sarcoplasmic reticulum Ca^2+^ ATPase and activating ryanodine receptors [303].

### 9.1. Spice Essential Oils in Postharvest Disease Mitigation

The composite solutions of aqueous extract of ginger, garlic and onion improve the shelf life (for about 5–6 days), anti-bacteria, antioxidation and sensory quality of stewed pork [304]. The addition in active packaging of ginger Eos extend the shelf life of poultry meat and meat products since they reduce lipid oxidation and microbiological growth [305,306]. Nanoemulsions of *Thymus daenensis* L. Eos are antibacterial, which can prolong stability and meliorate sensorial attributes in mayonnaise [307]. Garlic and ginger extracts improve the antioxidant activity, antimicrobial ability against some foodborne pathogens (e.g., *Bacillus subtilis* DB 100 host, *Escherichia coli* BA 12296, *Clostridium botulinum* ATCC 3584, *Staphylococcus aureus* NCTC10788 and *Salmonella senftenberg* ATCC 8400) and reduce thiobarbituric acid reactive substances levels, in herring fish fillet [305]. The nanoencapsulated form of the Garlic Eos is more effective than free form when incorporating into an active packaging (chitosan and whey protein films) to extend the shelf life of refrigerated vacuum-packed sausage [308].

### 9.2. Herb Essential Pils in Postharvest Disease Mitigation

The cumin and clove essential oils reduced Escherichia coli, Listeria monocytogenes, Salmonella, Campylobacter jejuni, Yersinia enterocolitica, Clostridium perfringens, Toxoplasma Gondi and Staphylococcus aureus bacterial cells in processed meat products [309].

Ethanolic extract of the *Cinnamomum zeylanicum* bark has a high antimicrobial effect against *Staphylococcus aureus*, *Escherichia coli* [310], *Listeria monocytogenes*, *Salmonella enterica*, food-borne pathogens [311].

Extracts of oregano and clove inhibit the lipid oxidation and reduce the *Listeria monocytogenes*, *Salmonella enterica* and *Staphylococcus aureus* numbers in cheese at room temperature [311].

The *Ocimum basilicum* L. Eos. have antifungal activity against *Candida albicans*, *Aspergillus niger* [312], *Aspergillus flavus* [313] and *Penicillium nalgiovense* [314]. The basil (*Ocimum basilicum*) leaves extract added to active film extends the shelf life of eggplant up to 16 days. It reserved eggplants’ moisture loss, retarded the improvement in total soluble solids, firmness and color changes [315]. Recently, Gundewadi et al. (2018) have shown a desirable inhibitory activity of basil Eos against fungi, *P. chrysogenum* and *A. flavus* when the nanoemulsions of the lipophilic active ingredients are dispersed in an aqueous media and *sapindus* extract serves as a surfactant for nano emulsification purposes [316].

Chitosan packages infused with *Origanum vulgare* essential oil maintain the grapes’ quality, physical and sensory attributes in post-harvest storage [317].

Eugenol and thymol extend strawberry shelf life by improving resistance to spoilage, deterioration and enhances their free radical scavenging capacity [318]. Eugenol and thymol reduce weight loss, skin color variation, ripening and decay of grape berries when used in a modified atmosphere package [319].

The fennel Eos addition to biodegradable film (based on polyhydroxybutyrate and polylactic acids) preserves oysters’ shelf-life improving the oxygen barrier performance, antioxidant activity and antimicrobial activity against aerobic and anaerobic bacteria [320]. Carvacrol, perillaldehyde and anethole improve total anthocyanins, phenolics and antioxidant activity, in blueberry fruit [321].

Postharvest Eos (e.g., linalool, perillaldehyde, cinnamaldehyde, anethole, cinnamic acid and carvacrol) treatments improve the antioxidant potential in raspberries with perillaldehyde [322].

### 9.3. Sauce Contribution in Postharvest Disease Mitigation

The soy sauce added to the Tambaqui fillet (processed by the sous vide) improves its shelflife [323].

### 9.4. Condiment Contribution in Postharvest Disease Mitigation

Vinegar prolongs the shelf life and palatability of common mackerel [324]. The powdered-buffered vinegar and liquid-buffered vinegar decrease the psychrotrophic growth of *Salmonella typhimurium* in ground beef patties [325]. During chilled storage, vinegar added to silver carp inhibit acid phosphatase (related to the freshness and flavor of fish) and alkaline phosphatase and enhances the accumulation of inosine monophosphate and free amino acids [326].

## 10. Food-to-Food Fortification

Food fortification aims to enhance people’s health. The main problem of classic food fortification depends on the economic problems related to food processing, especially in developing countries where the risk of malnutrition is very worrying. Food to food fortification is a new approach to food fortification. It uses the accessible local resource (animal or plant) to fortify another food [327]. Herbs, spices (essential oils, extract powder, fresh, etc.) and sauces are added to dairy products to improve their functional properties [323].

### 10.1. Spice’s Contribution to the Functional Properties of Foods

The addition of *Allium sativum* water extract during fermentation increases lactic acid bacteria in yogurt [328].

The supplement of cinnamon powder into yogurt enhances the antioxidant activity, the total phenolic content and phenolics bioaccessibility into the gastrointestinal tract [329]. The addition of ginger extracts into yogurt improves its antigenotoxic and antioxidant effects [330].

### 10.2. Herb’s Contribution to the Functional Properties of Foods

The supplement of basil into yogurt enhances the content of bioactive peptides and antioxidant properties [331]. The addition of dry basil leaves preserves and functionalizes cheeses. The basil leaves improve the cheese’s antioxidant activity, prevent the degradation of the protein (due to basil’s antimicrobial activity), fatty acids peroxidation and accelerating moisture loss [332]. The treatment of shredded iceberg lettuce with basil leaves’ extract positively affects its total phenolic content, antioxidant potential and no effect on consumer acceptability [333].

The fortify of cheese with dry rosemary and parsley improves cheese’s antioxidant properties [334].

The supplement of sage, or thyme and cumin essential oils to butter enhances its oxidative stability during storage time [335,336].

The addition of ginger into ice cream improves its total phenols and antioxidant activity [337].

### 10.3. Sauces Contribution to the Functional Properties of Foods

Soy sauce reduces lipid oxidation in meat products through the chelating activity of Fe^2+^ [338]. Soy sauce, Miso and fish sauces improve the *Z*-isomerization of lycopene in processed tomato products, enhancing the functionality of dishes since Z isomer is the most bioavailable [339].

### 10.4. Condiments Contribution to the Functional Properties of Foods

Extra virgin olive oil improves polyunsaturated fatty acids oxidative stability in algae oil. The EVOO’s MUFA decreased the n-3 algae’ PUFA and the EVOO’s secoiridoid reduced the algae’s triglyceride hydrolysis, simplifying their industry’s application [340].

## 11. Effect of Cooking on Spices, Condiments, Extra Virgin Olive Oil and Aromas

### 11.1. Effect of Cooking on Spices

The radical scavenging potential of ginger, garlic, cinnamon and turmeric depends on the cooking method. Microwaves decrease antioxidant activity. Instead, boiling and steaming increase it. Heat treatment also regulates their antimicrobial activity. Microwave, bake, grill and frying determine to lose their antimicrobial activity. Instead, boiled and steam methods decreased it [341]. The use of pepper onion, garlic, chili pepper, fennel and cumin, before grilling, frying, or roasting the meat prevents the formation of heterocyclic aromatic amines (Has) and polycyclic aromatic hydrocarbons (PAHs), compounds known to be associated with cancer development [342,343]. HAs are made during the cooking of protein-rich foods by the interaction between creatine/creatinine and free amino acids or with hexoses from the Maillard reaction [344]. The incomplete pyrolysis or combustion makes PAHs of organic matter. They are obtained by thermal degradation of fatty acids, triglycerides, steroids and amino acids [345]. The phenolics and organosulfur compounds in spices suppress the reactive species and/or interact on reactions, stopping byproducts’ formation [342]. Cooking methods and the fermentation process affect garlic antioxidant capacities. Raw garlic has more antioxidant activity than cooked garlic and black garlic (fermented garlic) has more significant antioxidant activity than crude garlic [346].

### 11.2. Effects of Cooking on Soy Sauce

Soy sauce preserves lipids from oxidation during cooking [347]. Phenolic compounds (by soybean) and antioxidative Maillard reaction products (by a non-enzymatic browning reaction) chelate ferrous ion a catalyst of lipid oxidation reaction [348].

### 11.3. Effects of Cooking on EVOO Degradation

EVOO has good thermal resistance in comparison with other vegetable oils. It is due to the high MUFA profile, phenolic composition and vitamin E. The initial olive oil composition, the period of the olive harvest and heating conditions (temperature, cooking process time and food presence) regulate the degradation rate and time required to degrade the antioxidant pool [338]. The absence of refining gave a high acidity to the EVOO and decreased its upper thermal limits in response to the released free fatty acids’ lower boiling point. Under frying and roasting conditions (180–190 °C), EVOO performance is better than other vegetable oils. It is quickly degraded under microwave processing, but food decreases the thermo-oxidative effects of microwave heating [338]. EVOO degradation and underwater boiling conditions, mainly depend on heating time, food and loss of phenolic compounds into the water phase. As a result, wherever possible, olive oil must be added more closely to the final cooking process [349].

## 12. Spices Side Effects

The consumption of some spices can cause side effects. For example, ginger can determine gastrointestinal (e.g., heartburn, diarrhea, bloating, gas, abdominal pain and epigastric distress), cardiovascular and respiratory symptoms [350]. High doses are not recommended in pregnancy, lactation and patient with bleeding disorders since it has anti-platelet property [351]. The most common garlic’s side effect is halitosis (bad breath) and body odor, especially when the raw form of the herbs is taken due to allyl methyl sulfide. A rare allergy to garlic was ascribed to protein allinase, which made hypersensitivity responses via immunoglobulin E [352]. Fenugreek ingestion can determine hypoglycemia in diabetics persons, diarrhea, abdominal distention, dyspepsia and flatulence [353]. Tumeric and curcumin can determine dermatitis and urticaria (immunoglobulin E mediated) especially following direct curcumin exposure to the skin or scalp [354]. Higher curcumin doses increase carcinogenesis, enhancing ROS cell levels [355].

## 13. Conclusions

The spices and condiments play an essential role in our diet that goes far beyond flavoring our dishes. They can be considered supplements of molecules functional to prevent oxidative stress injury, inflammation damage and chronic degenerative diseases that afflict our society, such as cardiovascular diseases and cancer. When multiple spices are used to prepare a dish, there is the possibility that they can have synergistic effects, increasing their health potential. However, more in-depth information on the effects of exposure to their bio components is needed to define intervention strategies to maximize beneficial effects and minimize unwanted side effects.

## Figures and Tables

**Table 1:** Class of flavonoids and their seasoning sources.

Class of Flavonoids	Chemical Structures	Condiments and/or Spices Sources
**Anthocyanin**	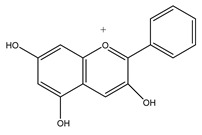	Wine vinegar
**Flavanones**	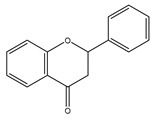	Soy sauce, parsley, chilli pepper.
**Flavanols**	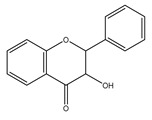	Fenugreek, curry, tumeric, garlic, ginger.
**Flavones**	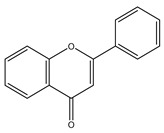	EVOO, soy sauce, fenugreek, basil, parsley.
**Flavonols**	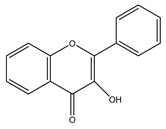	EVOO, fenugreek, curry, tumeric, garlic, ginger, basil, parsley, fennel, chilli pepper, sage.
**Isoflavonoids**	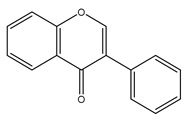	Fenugreek, soy sauce, Miso.
**Chalchones**	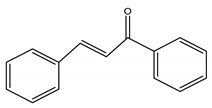	Tumeric

## References

[1] García-Casal M.N., Pena-Rosas J.P., Malave H.G. (2016). Sauces, spices, and condiments: Definitions, potential benefits, consumption patterns, and global markets. Ann. N. Y. Acad. Sci..

[2] Camarena D.M., Sanjuán A.I., Philippidis G. (2011). Influence of ethnocentrism and neophobia on ethnic food consumption in Spain. Appetite.

[3] Verbeke W., López G.P. (2005). Ethnic food attitudes and behaviour among Belgians and Hispanics living in Belgium. Br. Food J..

[4] Bell B., Adhikari K., Chambers E., Cherdchu P., Suwonsichon T. (2011). Ethnic food awareness and perceptions of consumers in Thailand and the United States. Nutr. Food Sci..

[5] Chung L., Chung S.J., Kim J.Y., Kim K.O., O’ Mahony M., Vickers Z., Cha S.M., Ishii R., Baures K., Kim H.R. (2012). Comparing the liking for Korean style salad dressings and beverages between US and Korean consumers: Effects of sensory and non-sensory factors. Food Qual. Prefer..

[6] Hong J.H., Lee K.W., Chung S.J., Chung L., Kim H.R., Kim K.O. (2012). Sensory characteristics and cross-cultural comparisons of consumer acceptability for *Gochujang* dressing. Food Sci. Biotechnol..

[7] Seasoning and Spices Market Size, Share & Trends Analysis Report by Product (Herbs, Salt & Salts Substitutes, Spices), by Application, by Region, and Segment Forecasts, 2020–2027. https://www.grandviewresearch.com/industry-analysis/seasonings-spices-market#:~:text=The%20global%20seasoning%20and%20spices%20market%20size%20was%20estimated%20at,USD%2015.44%20billion%20in%202020.

[8] Bhagya H.P., Raveendra Y.C., Lalithya K.A. (2015). Multi benificial uses of spices: A brief review. Acta Sci. Nutr. Health.

[9] Fanelli F., Cozzi G., Raiola A., Dini I., Mulè G., Logrieco A.F., Ritieni A. (2017). Raisins and Currants as Conventional Nutraceuticals in Italian Market: Natural Occurrence of Ochratoxin A. J. Food Sci..

[10] Dini I., Laneri S. (2019). Nutricosmetics: A brief overview. Phytother. Res..

[11] Laneri S., Di Lorenzo R.M., Bernardi A., Sacchi A., Dini I. (2020). *Aloe barbadensis*: A Plant of Nutricosmetic Interest. Nat. Prod. Commun..

[12] Laneri S., Di Lorenzo R., Sacchi A., Dini I. (2019). Dosage of bioactive molecules in the nutricosmeceutical *Helix aspersa muller* mucus and formulation of new cosmetic cream with moisturizing effect. Nat. Prod. Commun..

[13] Dini I., Holban A.M., Grumezescu A.M. (2018). Spices and herbs as therapeutic foods. Food Quality: Balancing Health and Disease.

[14] CODEX STAN 192-1995. 2013. Codex Alimentarius. Codex General Standard for Food Additives. Adopted in 1995. Revision 1997, 1999, 2001, 2003, 2004, 2005, 2006, 2007, 2008, 2009, 2010, 2011, 2012, 2013, 2014, 2015, 2016, 2017, 2018, 2019. http://www.fao.org/fao-who-codexalimentarius/sh-proxy/en/?lnk=1&url=https%253A%252F%252Fworkspace.fao.org%252Fsites%252Fcodex%252FStandards%252FCXS%2B192-1995%252FCXS_192e.pdf.

[15] Diniz do Nascimento L., Moraes A.A.B.d., Costa K.S.d., Pereira Galúcio J.M., Taube P.S., Costa C.M.L., Neves Cruz J., de Aguiar Andrade E.H., Faria L.J.G.d. (2020). Bioactive Natural Compounds and Antioxidant Activity of Essential Oils from Spice Plants: New Findings and Potential Applications. Biomolecules.

[16] Raj Singh L. (2019). Golden Shower: A wonder medicinal plant and its processing technology. Indian J. Appl. Res..

[17] Solorzano-Santos F., Miranda-Novales M.G. (2012). Essential oils from aromatic herbs as antimicrobial agents. Curr. Opin. Biotechnol..

[18] Farrell K.T. (1990). Spices, Condiments and Seasonings.

[19] Chadare F.J., Idohou R., Nago E., Affonfere M., Hounhouigan D.J., Agossadou J., Kévin T., Christel F., Sewanou K., Azokpota P. (2019). Conventional and food-to-food fortification: An appraisal of past practices and lessons learned. Food Sci. Nutr..

[20] WHO/FAO (2006). Guidelines on Food Fortification with Micronutrients.

[21] Klassen-Wigger P., Geraets M., Messier M.C., Detzel P., Lenoble H.P., Barclay D.V., Mannar M.G.V., Hurrell R.F. (2018). Micronutrient fortification of bouillon cubes in Central and West Africa. Food Fortification in a Globalized World.

[22] Mkambula P., Mbuya M.N.N., Rowe L.A., Sablah M., Friesen V.M., Chadha M., Osei A.K., Ringholz C., Vasta F.C., Gorstein J. (2020). The Unfinished Agenda for Food Fortification in Low- and Middle-Income Countries: Quantifying Progress, Gaps and Potential Opportunities. Nutrients.

[23] Dini I., Di Lorenzo R., Senatore A., Coppola D., Laneri S. (2020). Validation of Rapid Enzymatic Quantification of Acetic Acid in Vinegar on Automated Spectrophotometric System. Foods.

[24] Xia T., Zhang B., Duan W., Zhang J., Wang M. (2020). Nutrients and bioactive components from vinegar: A fermented and func-tionalfood. J. Funct. Foods.

[25] Cerezo A., Cuevas E., Winterhalter P., Garcia M., Troncoso A. (2010). Anthocyanin composition in Cabernet Sauvignon red wine vinegar obtained by submerged acetification. Food Res. Int. J..

[26] Kandylis P., Bekatorou A., Dimitrellou D., Plioni I., Giannopoulou K. (2021). Health Promoting Properties of Cereal Vinegars. Foods.

[27] Koulbanis C., Mellul M., Candau D. (1996). Cosmetic Composition Containing Vinegar as Active Anti-Ageing Agent, and Its Use in the Treatment of Dermatological Ageing. U.S. Patent.

[28] Servili M., Selvaggini  R., Esposto S., Taticchi A., Montedoro G., Morozzi G. (2004). Health and sensory properties of virgin olive oil hydrophilic phenols: Agronomic and technological aspect of production that affect their occurence in the oil. J. Chromatogr..

[29] Piroddi M., Albini A., Fabiani R., Giovannelli L., Luceri C., Natella F., Rosignoli P., Rossi T., Taticchi A., Servili M. (2017). Nutrigenomics of extra-virgin olive oil: A review. BioFactors.

[30] FoodData Central Search Results US Department of Agriculture (Agricultural Research Service) Fats and Oils, Oil, Olive, Extra Virgin. https://fdc.nal.usda.gov/fdc-app.html#/food-details/748608/nutrients.

[31] Angerosa F., Campestre C., Giansante L., Boskou D. (2006). Analysis and authentication. Olive Oil: Chemistry and Technology.

[32] Trade Standard Applying to Olive Oil and Olive-Pomace Oil. COI/T 15/NC No 3/Rev 7, May 2013. https://www.internationaloliveoil.org/wp-content/uploads/2019/11/COI-T.15-NC.-No-3-Rev.-13-2019-Eng.pdf.

[33] (2003). Codex Alimentarius. Codex Standard for Olive Oils, and Olive Pomace Oils, CODEX STAN 33-1981. Codex Alimentarius, Roma, Itália, Rev, 2. https://img.21food.cn/img/biaozhun/20100729/180/11294201.pdf.

[34] (1991). EC Regulation (1991). No. 2568/91/EEC, 1991. Off J Eur Commun L 248:1–83, July 11. https://eur-lex.europa.eu/eli/reg/1991/2568/2015-10-16.

[35] EC Regulation (2012). No. 432/2012 of 16 May 2012 Establishing a List of Permitted Health Claims Made on Foods, Other than Those Referring to the Reduction of Disease Risk and to Children’s Development and Health. *O.J.E.U.* L 136:1–40. http://data.europa.eu/eli/reg/2012/432/oj.

[36] EC Regulation (2013). No. 1332/2013 Amending Regulation (EU) No. 36/2012 Concerning Restrictive Measures in View of the Situation in Syria, 13 December 2013. OJ L, 335(14.12). http://data.europa.eu/eli/reg/2013/1332/oj.

[37] Kyçyk O., Aguilera M.P., Gaforio J.J. (2016). Sterol composition of virgin olive oil of forty-three olive cultivars from the World Collection Olive Germplasm Bank of Cordoba. J. Sci. Food Agric..

[38] Ghanbari R., Anwar F., Alkharfy K.M., Gilani A.-H., Saari N. (2012). Valuable Nutrients and Functional Bioactives in Different Parts of Olive (*Olea europaea* L.)—A Review. Int. J. Mol. Sci..

[39] Dini I., Graziani G., Fedele F.L., Sicari A., Vinale F., Castaldo L., Ritieni A. (2020). An environmentally friendly practice used in olive cultivation capable of increasing commercial interest in waste products from oil processing. Antioxidants.

[40] Dini I., Graziani G., Gaspari A., Fedele F.L., Sicari A., Vinale F., Cavallo P., Lorito M., Ritieni A. (2020). New strategies in the cultivation of olive trees and repercussions on the nutritional value of the extra virgin olive oil. Molecules.

[41] Dini I., Graziani G., Fedele F.L., Sicari A., Vinale F., Castaldo L., Ritieni A. (2020). Effects of *Trichoderma* Biostimulation on the Phenolic Profile of Extra-Virgin Olive Oil and Olive Oil By-Products. Antioxidants.

[42] Dini I., Marra R., Cavallo P., Pironti A., Sepe I., Troisi J., Scala G., Lombari P., Vinale F. (2021). *Trichoderma* Strains and Metabolites Selectively Increase the Production of Volatile Organic Compounds (VOCs) in Olive Trees. Metabolites.

[43] Dini I., Seccia S., Senatore A., Coppola D., Morelli E. (2020). Development and Validation of an Analytical Method for Total Polyphenols Quantification in Extra Virgin Olive Oils. Food Anal. Methods.

[44] Azzi A. (2019). Tocopherols, tocotrienols and tocomonoenols: Many similar molecules but only one vitamin E. Redox Biol..

[45] Ohara M., Lu H., Shiraki K., Ishimura Y., Uesaka T., Katoh O., Watanabe H. (2002). Prevention by long-term fermented miso of induction of colonic aberrant crypt foci by azoxymethane in F344 rats. Oncol. Rep..

[46] Watanabe H., Kashimoto N., Kajimura J., Kamiya K. (2006). A miso (Japanese soybean paste) diet conferred greater protection against hypertension than a sodium chloride diet in Dahl salt-sensitive rats. Hypertens. Res..

[47] Xu L., Du B., Xu B. (2015). A systematic, comparative study on the beneficial health components and antioxidant activities of commercially fermented soy products marketed in china. Food Chem..

[48] Chatterjee C., Gleddie S., Xiao C.-W. (2018). Soybean Bioactive Peptides and Their Functional Properties. Nutrients.

[49] FoodData Central Search Results US Department of Agriculture (Agricultural Research Service) Sauce, Ready-to-Serve, Soy Souce. https://fdc.nal.usda.gov/fdc-app.html#/food-details/1100456/nutrients.

[50] Li H., Zhao M., Su G., Lin L., Wang Y. (2016). Effect of soy sauce on serum uric acid levels in hyperuricemic rats and identification of flazin as a potent xanthine oxidase inhibitor. J. Agric. Food Chem..

[51] Kobayashi M., Watson R.R., Preedy V.R. (2013). Nutritional function of polysaccharides from soy sauce in the gastrointestinal tract. Bioactive Food as Dietary Interventions for Liver and Gastrointestinal Disease.

[52] Peng M., Liu J., Liu Z., Fu B., Hu Y., Zhou M., Fu C., Gao B., Wang C., Li D. (2018). Effect of citrus peel on phenolic compounds, organic acids and antioxidant activity of soy sauce. LWT-Food Sci. Technol..

[53] Watanabe H. (2013). Beneficial biological effects of miso with reference to radiation injury, cancer and hypertension. J. Toxicol. Pathol..

[54] Nakamura Y., Tsuji S., Tonogai Y. (2000). Determination of the levels of isoflavonoids in soybeans and soy-derived foods and estimation of isoflavonoids in the Japanese daily intake. J. AOAC Int..

[55] Minamiyama Y., Okada S., Farnworth E.R. (2008). Miso: Production, properties and benefits to heath. Handbook of Fermented Functional Foods.

[56] Sanlier N., Gökcen B.B., Sezgin A.C. (2019). Health benefits of fermented foods. Crit. Rev. Food Sci. Nutr..

[57] Takahashi M., Nagata M., Kaneko T., Suzuki T. (2020). Miso soup consumption enhances the bioavailability of the reduced form of supplemental coenzyme q(10). J. Nutr. Metab..

[58] Yoshikawa S., Kurihara H., Kawai Y., Yamazaki K., Tanaka A., Nishikiori T., Ohta T. (2010). Effect of halotolerant starter microorganisms on chemical characteristics of fermented Chum Salmon (*Oncorhynchus keta*) sauce. J. Agric. Food Chem..

[59] Giri A., Osako K., Ohshima T. (2010). Identification and characterisation of headspace volatiles of fish *miso*, a Japanese fish meat based fermented paste, with special emphasis on effect of fish species and meat washing. Food Chem..

[60] Gowda S.G.S., Narayan B., Gopal S. (2016). Bacteriological properties and health-related biochemical components of fermented fish sauce: Anoverview. Food Rev. Int..

[61] Sun J., Yu X., Fang B., Ma L., Xue C., Zhang Z., Mao X. (2016). Effect of fermentation by Aspergillus oryzaeon the biochemical and sensory proper-ties of anchovy (*Engraulis japonicus*) fish sauce. Int. J. Food Sci. Technol..

[62] Rhyu M.-R., Kim Y., Misaka T. (2021). Suppression of hTAS2R16 Signaling by Umami Substances. Int. J. Mol. Sci..

[63] Hamzeh A., Noisa P., Yongsawatdigul J. (2020). Characterization of the antioxidant and ACE-inhibitory activities of Thai fish sauce at different stages of fermentation. J. Funct. Foods.

[64] Li F., Liu W., Yamaki K., Liu Y., Fang Y., Li Z., Chem M., Wang C. (2016). Angiotensin I-converting enzyme inhibitory effect of chinese soypaste along fermentation and ripening: Contribution of early soybean protein borne peptides and late Maillard reaction products. Int. J. Food Prop..

[65] Lopetcharat K., Choi Y.J., Park J.W. (2001). Fish sauce products and manufacturing: A review. Food Rev. Int..

[66] FoodData Central Search Results U.S. Department of Agriculture (Agricultural Research Service) Sauce, Ready-to-Serve, Fish Sauce. https://fdc.nal.usda.gov/fdc-app.html#/food-details/1099270/nutrients.

[67] Dincer T., Cakli S., Kilinc B., Tolasa S. (2010). Amino acids and fatty acid composition content of fish sauce. J. Adv. Vet. Anim. Res..

[68] Han S.-C., Kang G.-J., Ko Y.-J., Kang H.-K., Moon S.-W., Ann Y.-S., Yoo E.-S. (2012). Fermented fish oil suppresses T helper 1/2 cell response in a mouse model of atopic dermatitis via generation of CD4+ CD25+ Foxp3+ T cells. BMC Immunol..

[69] Di Cagno R., Surico R.F., Paradiso A., De Angelis M., Salmon J.C., Buchin S., De Gara L., Gobbetti M. (2009). Effect of autochthonous lactic acid bacteria starters on health-promoting and sensory properties of tomato juices. Int. J. Food Microb..

[70] Cousin F.J., Le Guellec R., Schlusselhuber M., Dalmasso M., Laplace J.-M., Cretenet M. (2017). Microorganisms in fermented apple beverages: Current knowledge and future directions. Microorganisms.

[71] FoodData Central Search Results US Department of Agriculture (Agricultural Research Service) Sauce, Ready-to-Serve, Pepper, TABASCO. https://fdc.nal.usda.gov/fdc-app.html#/food-details/174528/nutrients.

[72] Farias V.L.D., Araújo Í.M.D.S., Rocha R.F.J.D., Garruti D.D.S., Pinto G.A.S. (2020). Enzymatic maceration of Tabasco pepper: Effect on the yield, chemical and sensory aspects of the sauce. LWT.

[73] Dhakal S., Chao K., Schmidt W., Qin J., Kim M., Huang Q. (2018). Detection of Azo Dyes in Curry Powder Using a 1064-nm Dispersive Point-Scan Raman System. Appl. Sci..

[74] (2020). FoodData Central Search Results.

[75] Ashokkumar K., Pandian A., Murugan M., Dhanya M.K., Sathyan T., Sivakumar P., Raj S., Warkentin T.D. (2020). Profiling bioactive flavonoids and carotenoids in select south Indian spices and nuts. Nat. Prod. Res..

[76] Reddy B.M., Dhanpal C.K., Lakshmi B.V.S. (2018). A review on curr y leaves (*Murray akoenigii): Versatile* multi-potential medicinal plant. Int. J. Adv. Pharm. Med. Bioallied Sci..

[77] Tachibana Y., Kikuzaki H., Lajis N.H., Nakatani N. (2001). Antioxidative activity of carbazoles from *Murraya koenigii* leaves. J. Agric. Food Chem..

[78] Jurenka J.S. (2009). Anti-inflammatory properties of curcumin, a major constituent of *Curcuma longa*: A review of preclinical and clinical research. Altern. Med. Rev..

[79] Irshad S., Muazzam A., Shahid Z., Dalrymple M.B. (2018). *Curcuma longa* (Turmeric): An auspicious spice for antibacterial, phytochemical and antioxidant activities. Pak. J. Pharm. Sci..

[80] (2020). FoodData Central Search Results.

[81] Omosa L.K., Midiwo J.O., Kuete V. (2012). Curcuma Longa.

[82] Venkata K.C., Bagchi D., Bishayee A. (2017). A small plant with big benefits: Fenugreek (*Trigonella foenum-graecum* Linn.) for disease prevention and health promotion. Mol. Nutr. Food Res..

[83] Jacob B., Narendhirakannan R.T. (2019). Role of medicinal plants in the management of diabetes mellitus: A review. Biotechnol. J..

[84] (2019). FoodData Central Search Results.

[85] Diretto G., Rubio-Moraga A., Argandona J., Castillo P., Gomez-Gomez L., Ahrazem O. (2017). Tissue-specific accumulation of sulfur compounds and saponins in different parts of garlic cloves from purple and white ecotypes. Molecules.

[86] Szychowski K.A., Rybczynska-Tkaczyk K., Gawel-Beben K., Swieca M., Karas M., Jakubczyk A., Matysiak M., Binduga U.E., Gminski J. (2018). Characterization of active compounds of different garlic (*Allium sativum* L.) cultivars. Pol. J. Food Nutr. Sci..

[87] Bradley J.M., Organ C.L., Lefer D.J. (2016). Garlic-derived organic polysulfides and myocardial protection. J. Nutr..

[88] Wang Y.C., Guan M., Zhao X., Li X.L. (2018). Effects of garlic polysaccharide on alcoholic liver fibrosis and intestinal microflora in mice. Pharm. Biol..

[89] Nagella P., Thiruvengadam M., Ahmad A., Yoon J.Y., Chung I.M. (2014). Composition of polyphenols and antioxidant activity of garlic bulbs collected from different locations of Korea. Asian J. Chem..

[90] Shang A., Cao S.-Y., Xu X.-Y., Gan R.-Y., Tang G.-Y., Corke H., Mavumengwana V., Li H.-B. (2019). Bioactive Compounds and Biological Functions of Garlic (*Allium sativum* L.). Foods.

[91] Liu J., Guo W., Yang M.L., Liu L.X., Huang S.X., Tao L., Zhang F., Liu Y.S. (2018). Investigation of the dynamic changes in the chemical constituents of chinese “laba” garlic during traditional processing. RSC Adv..

[92] Kang J.S., Kim S.O., Kim G.Y., Hwang H.J., Kim B.W., Chang Y.C., Kim W.J., Kim C.M., Yoo Y.H., Choi Y.H. (2016). An exploration of the antioxidant effects of garlic saponins in mouse-derived C2C12 myoblasts. Int. J. Mol. Med..

[93] Park S.Y., Seetharaman R., Ko M.J., Kim D.Y., Kim T.H., Yoon M.K., Kwak J.H., Lee S.J., Bae Y.S., Choi Y.W. (2014). Ethyl linoleate from garlic attenuates lipopolysaccharide-induced pro-inflammatory cytokine production by inducing heme oxygenase-1 in RAW 264.7 cells. Int. Immunopharmacol..

[94] Rabe S.Z.T., Ghazanfari T., Siadat Z., Rastin M., Rabe S.Z.T., Mahmoudi M. (2015). Anti-inflammatory effect of garlic 14-kDa protein on LPS-stimulated-J774A.1 macrophages. Immunopharmacol. Immunotoxicol..

[95] Li M., Yan Y.X., Yu Q.T., Deng Y., Wu D.T., Wang Y., Ge Y.Z., Li S.P., Zhao J. (2017). Comparison of immunomodulatory effects of fresh garlic and black garlic polysaccharides on RAW 264. 7 macrophages. J. Food Sci..

[96] Asdaq S.M., Inamdar M.N. (2010). Potential of garlic and its active constituent, S-allyl cysteine, as antihypertensive and cardioprotective in presence of captopril. Phytomedicine.

[97] Leung A.Y., Leug A.Y. (1984). Chinese Herbal Remedies.

[98] Tang W., Eisenbrand G. (1992). Chinese Drugs of Plant Origin. Chemistry, Pharmacology, and Use in Traditional and Modern Medicine.

[99] Shirin Adel P.R., Prakash J. (2010). Chemical composition and antioxidant properties of ginger root (Zingiber officinale). J. Med. Plants Res..

[100] (2017). FoodData Central Search Results.

[101] Bhatt N., Waly M.I., Essa M.M., Ali A. (2013). Ginger: A functional herb. Food as Medicine.

[102] De Krishna A., Minakshi D., Watson R.R., Singh R.B., Takahashi T. (2019). Functional and therapeutic applications of some important spices. The Role of Functional Food Security in Global Health.

[103] Mohd Hassan N., Yusof N.A., Yahaya A.F., Mohd Rozali N.N., Othman R. (2019). Carotenoids of *Capsicum* Fruits: Pigment Profile and Health-Promoting Functional Attributes. Antioxidants.

[104] Shahrajabian M.H., Sun W., Cheng Q. (2020). Chemical components and pharmacological benefits of basil (Ocimum basilicum): A review. Int. J. Food Propert..

[105] Ciesielski W., Gąstoł M., Kulawik D., Oszczęda Z., Pisulewska E., Tomasik P. (2020). Specific Controlling Essential Oil Composition of Basil (*Ocimum basilicum* L.) Involving Low-Temperature, Low-Pressure Glow Plasma of Low Frequency. Water.

[106] Zhan Y., An X., Wang S., Sun M., Zhou H. (2020). Basil polysaccharides: A review on extraction, bioactivities and pharmacological applications. Bioorg. Med. Chem..

[107] Slimestad R., Fossen T., Brede C. (2020). Flavonoids and other phenolics in herbs commonly used in Norwegian commercial kitchens. Food Chem..

[108] Dorman H.J., Lantto T.A., Raasmaja A., Hiltunen R. (2011). Antioxidant, pro-oxidant and cytotoxic properties of parsley. Food Funct..

[109] Barros L., Carvalho A.M., Ferreira I.C.F.R. (2010). The nutritional composition of fennel (*Foeniculum vulgare*): Shoots, leaves, stems and inflorescences. LWT Food Sci. Technol..

[110] Ferioli F., Giambanelli E., D’Antuono L.F. (2017). Fennel (*Foeniculum vulgare* Mill. Subsp. *piperitum*) florets, a traditional culinary spice in Italy: Evaluation of phenolics and volatiles in local populations, and comparison with the composition of other plant parts. J. Sci. Food Agric..

[111] FoodData Central Search Results US Department of Agriculture (Agricultural Research Service) Sage. https://fdc.nal.usda.gov/fdc-app.html#/food-details/170935/nutrients.

[112] Ben Khedher M.R., Khedher S.B., Chaieb I., Tounsi S., Hammami M. (2017). Chemical composition and biological activities of Salvia officinalis essential oil from Tunisia. EXCLI J..

[113] Ghorbani A., Esmaeilizadeh M. (2017). Pharmacological properties of *Salvia officinalis* and its components. J. Tradit. Complement. Med..

[114] De Caro C., Raucci F., Saviano A., Cristiano C., Casillo G.M., Di Lorenzo R., Sacchi A., Laneri S., Dini I., De Vita S. (2020). Pharmacological and molecular docking assessment of cryptotanshinone as natural-derived analgesic compound. Biomed. Pharmacother..

[115] Radi R., Beckman J.S., Bush K.M., Freeman B.A. (2013). Peroxynitrite, a stealthy biological oxidant. Int. J. Biol. Chem..

[116] Rehman K., Akash M.S.H. (2017). Mechanism of generation of oxidative stress and pathophysiology of type 2 diabetes mellitus: How are they interlinked?. J. Cell Biochem..

[117] Lobo V., Patil A., Phatak A., Chandra N. (2010). Free radicals, antioxidants and functional foods: Impact on human health. Pharmacogn. Rev..

[118] Ito F., Sono Y., Ito T. (2019). Measurement and Clinical Significance of Lipid Peroxidation as a Biomarker of Oxidative Stress: Oxidative Stress in Diabetes, Atherosclerosis, and Chronic Inflammation. Antioxidants.

[119] Lichtenberg D., Pinchuk I. (2015). Oxidative stress, the term and the concept. Biochem. Biophys. Res. Commun..

[120] Dotan Y., Lichtenberg D., Pinchuk I. (2004). Lipid peroxidation cannot be used as a universal criterion of oxidative stress. Prog. Lipid Res..

[121] Guo W., Adachi T., Matsui R., Xu S., Jiang B., Zou M.H., Kirber M., Lieberthal W., Cohen R.A. (2003). Quantitative assessment of tyrosine nitration of manganese superoxide dismutase in angiotensin II-infused rat kidney. Am. J. Physiol. Heart Circ. Physiol..

[122] Khan J., Brennand D.M., Bradley N., Gao B., Bruckdorfer R., Jacobs M. (1998). 3-Nitrotyrosine in the proteins of human plasma determined by an ELISA method. Biochem. J..

[123] Parkin J., Cohen B. (2001). An overview of the immune system. Lancet.

[124] Calder P.C. (2020). Nutrition, immunity and COVID-19. BMJ Nutr. Prev. Health.

[125] Gombart A.F., Pierre A., Maggini S. (2020). A Review of Micronutrients and the Immune System–Working in Harmony to Reduce the Risk of Infection. Nutrients.

[126] Murphy K., Weaver C. (2016). Janeway’s Immunobiology.

[127] Hengartner H., Odermatt B., Schneider R., Schreyer M., Walle G., MacDonald H.R., Zinkernagel R.M. (1988). Deletion of self-reactive T cells before entry into the thymus medulla. Nature.

[128] Jenkinson E.J., Kingston R., Smith C.A., Williams G.T., Owen J.J. (1989). Antigen-induced apoptosis in developing T cells: A mechanism for negative selection of the T cell receptor repertoire. Eur. J. Immunol..

[129] Pawelec G., Larbi A., Derhovanessian E. (2010). Senescence of the human immune system. J. Comp. Pathol..

[130] Pera A., Campos C., Lopez N., Hassouneh F., Alonso C., Tarazona R., Solana R. (2015). Immunosenescence: Implications for response to infection and vaccination in older people. Maturitas.

[131] Agarwal S., Busse P.J. (2010). Innate and adaptive immunosenescence. Ann. Allergy Asthma Immunol..

[132] Montgomery R.R., Shaw A.C. (2015). Paradoxical changes in innate immunity in aging: Recent progress and new directions. J. Leukoc. Biol..

[133] Ventura M.T., Casciaro M., Gangemi S., Buquicchio R. (2017). Immunosenescence in aging: Between immune cells depletion and cytokines up-regulation. Clin. Mol. Allergy.

[134] Calder P.C., Bosco N., Bourdet-Sicard R., Capuron L., Delzenne N., Doré J., Franceschi C., Lehtinen M.J., Recker T., Salvioli S. (2017). Health relevance of the modification of low-grade inflammation in ageing (inflammageing) and the role of nutrition. Ageing Res. Rev..

[135] Bennett M., Gilroy D.W. (2017). Lipid mediators in inflammation. Myeloid cells in health and disease: A synthesis.

[136] Honce R., Schultz-Cherry S. (2019). Impact of obesity on influenza A virus pathogenesis, immune response, and evolution. Front. Immunol..

[137] Frasca D., Diaz A., Romero M., Blomberg B.B. (2017). Ageing and obesity similarly impair antibody responses. Clin. Exp. Immunol..

[138] O’Shea D., Hogan A.E. (2019). Dysregulation of natural killer cells in obesity. Cancers.

[139] Huttunen R., Syrjanen J. (2013). Obesity and the risk and outcome of infection. Int. J. Obes..

[140] Bogden J.D., Oleske J.M. (2007). The essential trace minerals, immunity, and progression of HIV-1 infection. Nutr. Res..

[141] Kris-Etherton P.M., Harris W.S., Appel L.J. (2002). Fish consumption, fish oil, omega-3 fatty acids, and cardiovascular disease. Circulation.

[142] Huang C., Wang Y., Li X., Ren L., Zhao J., Hu Y., Zhang L., Fan G., Xu J., Gu X. (2020). Clinical features of patients infected with 2019 novel coronavirus in Wuhan, China. Lancet.

[143] Bermudez B., Lopez S., Ortega A., Varela L.M., Pacheco Y.M. (2011). Oleic acid in olive oil: From a metabolic framework toward a clinical perspective. Curr. Pharm. Des..

[144] Schwingshack L., Hoffmann G. (2012). Monounsaturated fatty acids and risk of cardiovascular disease: Synopsis of the evidence available from systematic reviews and meta-analyses. Nutrients.

[145] Lehninger A.L., Nelson D.L., Cox M.M. (2005). Principles of Biochemistry.

[146] Patterson E., Wall R., Fitzgerald G.F., Ross R.P., Stanton C. (2012). Health implications of high dietary omega-6 polyunsaturated fatty acids. J. Nutr. Metab..

[147] Food and Nutrition Tips during Self-Quarantine. https://www.euro.who.int/en/health-topics/health-emergencies/coronavirus-covid-19/publications-and-technical-guidance/food-and-nutrition-tips-during-self-quarantine.

[148] Abu-Farha M., Thanaraj T.A., Qaddoumi M.G., Hashem A., Abubaker J., Al-Mulla F. (2020). The Role of Lipid Metabolism in COVID-19 Virus Infection and as a Drug Target. Int. J. Mol. Sci..

[149] Waitzberg D.L., Torrinhas R.S. (2009). Fish oil lipid emulsions and immune response: What clinicians need to know. Nutr. Clin. Pract..

[150] Saini R.K., Keum Y.S. (2018). Omega-3 and omega-6 polyunsaturated fatty acids: Dietary sources, metabolism, and significance—A review. Life Sci..

[151] Fredman G., Serhan C.N. (2011). Specialized proresolving mediator targets for RvE1 and RvD1 in peripheral blood and mechanisms of resolution. Biochem. J..

[152] Spite M., Norling L.V., Summers L., Yang R., Cooper D., Petasis N.A., Flower J., Perretti M., Serhan C.N. (2009). Resolvin D2 is a potent regulator of leukocytes and controls microbial sepsis. Nature.

[153] Hasturk H., Kantarci A., Goguet-Surmenian E., Blackwood A., Andry C., Serhan C.N., Van Dyke T.E. (2007). Resolvin E1 regulates inflammation at the cellular and tissue level and restores tissue homeostasis in vivo. J. Immunol..

[154] Titos E., Rius B., González-Périz A., López-Vicario C., Morán-Salvador E., Martínez-Clemente M., Arroyo V., Claria J. (2011). Resolvin D1 and Its Precursor Docosahexaenoic Acid Promote Resolution of Adipose Tissue Inflammation by Eliciting Macrophage Polarization toward an M2-Like. Phenotype. J. Immunol..

[155] Lythgoe M.P., Middleton P. (2020). Ongoing Clinical Trials for the Management of the COVID-19 Pandemic. Trends Pharmacol. Sci..

[156] Huo Q., Li B., Cheng L., Wu T., You P., Shen S., Li Y., He Y., Tian W., Li R. (2019). Dietary Supplementation of Lysophospholipids Affects Feed Digestion in Lambs. Animals.

[157] Johnson E.J., Russell R.M., Coates P.M., Betz J.M., Blackman M.R. (2010). Beta-Carotene. Encyclopedia of Dietary Supplements.

[158] Ross C.A., Vitamin A., Coates P.M., Betz J.M., Blackman M.R. (2010). Encyclopedia of Dietary Supplements.

[159] Abdelhamid L., Luo X.M. (2018). Retinoic Acid, Leaky Gut, and Autoimmune Diseases. Nutrients.

[160] Maggini S., Beveridge S., Sorbara J.P., Senatore G. (2008). Feeding the immune system: The role of micronutrients in restoring resistance to infections. CAB Rev..

[161] Biesalski H.K. (2016). Nutrition meets the microbiome: Micronutrients and the microbiota. Ann. N. Y. Acad. Sci..

[162] Levy M., Thaiss C.A., Elinav E. (2016). Metabolites: Messengers between the microbiota and the immune system. Genes Dev..

[163] Sirisinha S. (2015). The pleiotropic role of vitamin A in regulating mucosal immunity. Asian Pac. J. Allergy Immunol..

[164] Chew B.P., Park J.S. (2004). Carotenoid action on the immune response. J. Nutr..

[165] Solomons N.W., Vitamin A., Bowman B., Russell R. (2006). Present Knowledge in Nutrition.

[166] Mccullough F.S., Northropclewes C.A., Thurnham D.I. (1999). The effect of vitamin A on epithelial integrity. Proc. Nutr. Soc..

[167] Wang J.L., Swartz-Basile D.A., Rubin D.C., Levin M.S. (1997). Retinoic acid stimulates early cellular proliferation in the adapting remnant rat small intestine after partial resection. J. Nutr..

[168] Riabroy N., Tanumihardjo S.A. (2014). Oral doses of retinyl ester track chylomicron uptake and distribution of vitamin A in a male piglet model for newborn infants. J. Nutr..

[169] Kiss I., Rühl R., Szegezdi E., Fritzsche B., Toth B., Pongrácz J., Perlmann T., Fésüs L., Szondy Z. (2008). Retinoid receptor-activating ligands are produced within the mouse thymus during postnatal development. Eur. J. Immunol..

[170] Kuwata T., Wang I.M., Tamura T., Ponnamperuma R.M., Levine R., Holmes K.L., Morse H.C., De Luca L.M., Ozato K. (2000). Vitamin A deficiency in mice causes a systemic expansion of myeloid cells. Blood.

[171] Chang H.K., Hou W.S. (2015). Retinoic acid modulates interferon- production by hepatic natural killer T cells via phosphatase 2A and the extracellular signal-regulated kinase pathway. J. Interferon Cytokine Res..

[172] Wynn T.A., Vannella K.M. (2016). Macrophages in Tissue Repair, Regeneration, and Fibrosis. Immunity.

[173] Ross A.C. (1996). Vitamin A deficiency and retinoid repletion regulate the antibody response to bacterial antigens and the maintenance of natural killer cells. J. Clin. Immunol. Immunopathol..

[174] Hu N., Li Q.B., Zou S. (2018). Effect of vitamin A as an adjuvant therapy for pneumonia in children: A Meta-analysis. Zhongguo Dang dai er ke za zhi = Chin. J. Contemp. Pediatrics.

[175] Chowdhury A.I. (2020). Role and Effects of Micronutrients Supplementation in Immune System and SARS-Cov-2(COVID-19). Asian Pac. J. Allergy Immun..

[176] Hosomi K., Kunisawa J. (2017). The specific roles of vitamins in the regulation of immunosurveillance and maintenance of immunologic homeostasis in the gut. Immune Netw..

[177] Ross A., Shils M., Shike M., Ross A., Caballero B., Cousins R. (2006). Vitamin A and Carotenoids. Modern Nutrition in Health and Disease.

[178] Yoshii K., Hosomi K., Sawane K., Kunisawa J. (2019). Metabolism of Dietary and Microbial Vitamin B Family in the Regulation of Host Immunity. Front. Nutr..

[179] Luna G., Alping P., Burman J., Fink K., Fogdell-Hahn A., Gunnarsson M., Hillert J., Langer-Gould A., Lycke J., Nilsson P. (2019). Infection Risks Among Patients with Multiple Sclerosis Treated With Fingolimod, Natalizumab, Rituximab, and Injectable Therapies. JAMA Neurol..

[180] Villarruz-Sulit M.V., Cabaluna I.T. (2020). Should, B Vitamins be used in the treatment of COVID-19?. Asia Pac. Cent. Evid. Based Healthcare.

[181] Kant A.K., Block G. (1990). Dietary vitamin B-6 intake and food sources in the US population: NHANES II,1976–1980. Am. J. Clin. Nutr..

[182] Watanabe F. (2007). Vitamin B12 sources and bioavailability. Exp. Biol. Med..

[183] Burns J.J. (1957). Missing step in man, monkey and guinea pig required for the biosynthesis of L-ascorbic acid. Nature.

[184] Nishikimi M., Fukuyama R., Minoshima S., Shimizu N., Yagi K. (1994). Cloning and chromosomal mapping of the human nonfunctional gene for L-gulono-gamma-lactone oxidase, the enzyme for L-ascorbic acid biosynthesis missing in man. J. Biol. Chem..

[185] Carr A., Frei B. (1999). Does vitamin C act as a pro-oxidant under physiological conditions?. FASEB J..

[186] Mandl J., Szarka A., Banhegyi G. (2009). Vitamin C: Update on physiology and pharmacology. Br. J. Pharmacol..

[187] Englard S., Seifter S. (1986). The biochemical functions of ascorbic acid. Annu. Rev. Nutr..

[188] Kivirikko K.I., Myllyla R., Pihlajaniemi T. (1989). Protein hydroxylation: Prolyl 4-hydroxylase, an enzyme with four cosubstrates and a multifunctional subunit. FASEB J..

[189] Geesin J.C., Darr D., Kaufman R., Murad S., Pinnell S.R. (1988). Ascorbic acid specifically increases type I and type III procollagen messenger RNA levels in human skin fibroblast. J. Investig. Dermatol..

[190] Kishimoto Y., Saito N., Kurita K., Shimokado K., Maruyama N., Ishigami A. (2013). Ascorbic acid enhances the expression of type 1 and type 4 collagen and SVCT2 in cultured human skin fibroblasts. Biochem. Biophys. Res. Commun..

[191] Nusgens B.V., Humbert P., Rougier A., Colige A.C., Haftek M., Lambert C.A., Richard A., Creidi P., Lapiere C.M. (2001). Topically applied vitamin C enhances the mRNA level of collagens I and III, their processing enzymes and tissue inhibitor of matrix metalloproteinase 1 in the human dermis. J. Investig. Dermatol..

[192] Tajima S., Pinnell S.R. (1996). Ascorbic acid preferentially enhances type I and III collagen gene transcription in human skin fibroblasts. J. Dermatol. Sci..

[193] Davidson J.M., Lu Valle P.A., Zoia O., Quaglino D., Giro M. (1997). Ascorbate differentially regulates elastin and collagen biosynthesis in vascular smooth muscle cells and skin fibroblasts by pretranslational mechanisms. J. Biol. Chem..

[194] Carr A., Maggini S. (2017). Vitamin C and immune function. Nutrients.

[195] Stankova L., Gerhardt N.B., Nagel L., Bigley R.H. (1975). Ascorbate and phagocyte function. Infect. Immunol..

[196] Winterbourn C.C., Vissers M.C. (1983). Changes in ascorbate levels on stimulation of human neutrophils. Biochim. Biophys. Acta.

[197] Parker A., Cuddihy S.L., Son T.G., Vissers M.C., Winterbourn C.C. (2011). Roles of superoxide and myeloperoxidase in ascorbate oxidation in stimulated neutrophils and H(2)O(2)-treated HL60 cells. Free Radic. Biol. Med..

[198] Oberritter H., Glatthaar B., Moser U., Schmidt K.H. (1986). Effect of functional stimulation on ascorbate content in phagocytes under physiological and pathological conditions. Int. Arch. Allergy Appl. Immunol..

[199] Goldschmidt M.C. (1991). Reduced bactericidal activity in neutrophils from scorbutic animals and the effect of ascorbic acid on these target bacteria in vivo and in vitro. Am. J. Clin. Nutr..

[200] Goldschmidt M.C., Masin W.J., Brown L.R., Wyde P.R. (1988). The effect of ascorbic acid deficiency on leukocyte phagocytosis and killing of actinomyces viscosus. Int. J. Vitam. Nutr. Res..

[201] Johnston C.S., Huang S.N. (1991). Effect of ascorbic acid nutriture on blood histamine and neutrophil chemotaxis in guinea pigs. J. Nutr..

[202] Rebora A., Dallegri F., Patrone F. (1980). Neutrophil dysfunction and repeated infections: Influence of levamisole and ascorbic acid. Br. J. Dermatol..

[203] Patrone F., Dallegri F., Bonvini E., Minervini F., Sacchetti C. (1982). Disorders of neutrophil function in children with recurrent pyogenic infections. Med. Microbiol. Immunol..

[204] Boura P., Tsapas G., Papadopoulou A., Magoula I., Kountouras G. (1989). Monocyte locomotion in anergic chronic brucellosis patients: The in vivo effect of ascorbic acid. Immunopharmacol. Immunotoxicol..

[205] Anderson R., Theron A. (1979). Effects of ascorbate on leucocytes: Part III. In vitro and in vivo stimulation of abnormal neutrophil motility by ascorbate. S. Afr. Med. J..

[206] Johnston C.S., Martin L.J., Cai X. (1992). Antihistamine effect of supplemental ascorbic acid and neutrophil chemotaxis. J. Am. Coll. Nutr..

[207] Anderson R., Oosthuizen R., Maritz R., Theron A., Van Rensburg A.J. (1980). The effects of increasing weekly doses of ascorbate on certain cellular and humoral immune functions in normal volunteers. Am. J. Clin. Nutr..

[208] Anderson R. (1981). Ascorbate-mediated stimulation of neutrophil motility and lymphocyte transformation by inhibition of the peroxidase/H_2_O_2_/halide system in vitro and in vivo. Am. J. Clin. Nutr..

[209] Ganguly R., Durieux M.F., Waldman R.H. (1976). Macrophage function in vitamin C-deficient guinea pigs. Am. J. Clin. Nutr..

[210] Corberand J., Nguyen F., Fraysse B., Enjalbert L. (1982). Malignant external otitis and polymorphonuclear leukocyte migration impairment. Improvement with ascorbic acid. Arch. Otolaryngol..

[211] Levy R., Schlaeffer F. (1993). Successful treatment of a patient with recurrent furunculosis by vitamin C: Improvement of clinical course and of impaired neutrophil functions. Int. J. Dermatol..

[212] Levy R., Shriker O., Porath A., Riesenberg K., Schlaeffer F. (1996). Vitamin C for the treatment of recurrent furunculosis in patients with imparied neutrophil functions. J. Infect. Dis..

[213] Nungester W.J., Ames A.M. (1948). The relationship between ascorbic acid and phagocytic activity. J. Infect. Dis..

[214] Shilotri P.G. (1977). Phagocytosis and leukocyte enzymes in ascorbic acid deficient guinea pigs. J. Nutr..

[215] Collins J.F., Ross A.C., Caballero B., Cousins R.J., Tucker K.L., Ziegler T.R. (2014). Modern Nutrition in Health and Disease.

[216] Prohaska J.R., Erdman J.W., Macdonald I.A., Zeisel S.H. (2012). Copper. Present Knowledge in Nutrition.

[217] Shilotri P.G. (1977). Glycolytic, hexose monophosphate shunt and bactericidal activities of leukocytes in ascorbic acid deficient guinea pigs. J. Nutr..

[218] Sharma P., Raghavan S.A., Saini R., Dikshit M. (2004). Ascorbate-mediated enhancement of reactive oxygen species generation from polymorphonuclear leukocytes: Modulatory effect of nitric oxide. J. Leukoc. Biol..

[219] Rebora A., Crovato F., Dallegri F., Patrone F. (1980). Repeated staphylococcal pyoderma in two siblings with defective neutrophil bacterial killing. Dermatologica.

[220] Vissers M.C., Wilkie R.P. (2007). Ascorbate deficiency results in impaired neutrophil apoptosis and clearance and is associated with up-regulation of hypoxia-inducible factor 1alpha. J. Leukoc. Biol..

[221] Fisher B.J., Kraskauskas D., Martin E.J., Farkas D., Wegelin J.A., Brophy D., Ward K.R., Voelkel N.F., Fowler A.A., Natarajan R. (2012). Mechanisms of attenuation of abdominal sepsis induced acute lung injury by ascorbic acid. Am. J. Physiol. Lung Cell. Mol. Physiol..

[222] Huijskens M.J., Walczak M., Koller N., Briede J.J., Senden-Gijsbers B.L., Schnijderberg M.C., Bos G.M., Germeraad W.T. (2014). Technical advance: Ascorbic acid induces development of double-positive T cells from human hematopoietic stem cells in the absence of stromal cells. J. Leukoc. Biol..

[223] Molina N., Morandi A.C., Bolin A.P., Otton R. (2014). Comparative effect of fucoxanthin and vitamin C on oxidative and functional parameters of human lymphocytes. Int. Immunopharmacol..

[224] Tanaka M., Muto N., Gohda E., Yamamoto I. (1994). Enhancement by ascorbic acid 2-glucoside or repeated additions of ascorbate of mitogen-induced IgM and IgG productions by human peripheral blood lymphocytes. Jpn. J. Pharmacol..

[225] Manning J., Mitchell B., Appadurai D.A., Shakya A., Pierce L.J., Wang H., Nganga V., Swanson P.C., May J.M., Tantin D. (2013). Vitamin C promotes maturation of T-cells. Antioxid. Redox Signal..

[226] Kennes B., Dumont I., Brohee D., Hubert C., Neve P. (1983). Effect of vitamin C supplements on cell-mediated immunity in old people. Gerontology.

[227] Anderson R., Hay I., van Wyk H., Oosthuizen R., Theron A. (1980). The effect of ascorbate on cellular humoral immunity in asthmatic children. S. Afr. Med. J..

[228] Fraser R.C., Pavlovic S., Kurahara C.G., Murata A., Peterson N.S., Taylor K.B., Feigen G.A. (1980). The effect of variations in vitamin C intake on the cellular immune response of guinea pigs. Am. J. Clin. Nutr..

[229] Feigen G.A., Smith B.H., Dix C.E., Flynn C.J., Peterson N.S., Rosenberg L.T., Pavlovic S., Leibovitz B. (1982). Enhancement of antibody production and protection against systemic anaphylaxis by large doses of vitamin C. Res. Commun. Chem. Pathol. Pharmacol..

[230] Prinz W., Bloch J., Gilich G., Mitchell G. (1980). A systematic study of the effect of vitamin C supplementation on the humoral immune response in ascorbate-dependent mammals. I. The antibody response to sheep red blood cells (a T-dependent antigen) in guinea pigs. Int. J. Vitam. Nutr. Res..

[231] Prinz W., Bortz R., Bregin B., Hersch M. (1977). The effect of ascorbic acid supplementation on some parameters of the human immunological defence system. Int. J. Vitam. Nutr. Res..

[232] Mohammed B.M., Fisher B.J., Kraskauskas D., Farkas D., Brophy D.F., Fowler A.A., Natarajan R. (2013). Vitamin C: A novel regulator of neutrophil extracellular trap formation. Nutrients.

[233] Chen Y., Luo G., Yuan J., Wang Y., Yang X., Wang X., Li G., Liu Z., Zhong N. (2014). Vitamin C mitigates oxidative stress and tumor necrosis factor-alpha in severe community-acquired pneumonia and LPS-induced macrophages. Mediat. Inflamm..

[234] Jeng K.C., Yang C.S., Siu W.Y., Tsai Y.S., Liao W.J., Kuo J.S. (1996). Supplementation with vitamins C and E enhances cytokine production by peripheral blood mononuclear cells in healthy adults. Am. J. Clin. Nutr..

[235] Kim Y., Kim H., Bae S., Choi J., Lim S.Y., Lee N., Kong J.M., Hwang Y.I., Kang J.S., Lee W.J. (2013). Vitamin C is an essential factor on the antiviral immune responses through the production of interferon-a/b at the initial stage of influenza A virus (H3N2) infection. Immune Netw..

[236] Gao Y.L., Lu B., Zhai J.H., Liu Y.C., Qi H.X., Yao Y., Chai Y.F., Shou S.T. (2017). The parenteral vitamin C improves sepsis and sepsis-induced multiple organ dysfunction syndrome via preventing cellular immunosuppression. Mediat. Inflamm..

[237] Portugal C.C., Socodato R., Canedo T., Silva C.M., Martins T., Coreixas V.S., Loiola E.C., Gess B., Rohr D., Santiago A.R. (2017). Caveolin-1-mediated internalization of the vitamin C transporter SVCT2 in microglia triggers an inflammatory phenotype. Sci. Signal..

[238] Dahl H., Degre M. (1976). The effect of ascorbic acid on production of human interferon and the antiviral activity in vitro. Acta Pathol. Microbiol. Scand. B.

[239] Karpinska T., Kawecki Z., Kandefer-Szerszen M. (1982). The influence of ultraviolet irradiation, L-ascorbic acid and calcium chloride on the induction of interferon in human embryo fibroblasts. Arch. Immunol. Ther. Exp..

[240] Siegel B.V. (1975). Enhancement of interferon production by poly(rI)-poly(rC) in mouse cell cultures by ascorbic acid. Nature.

[241] Canali R., Natarelli L., Leoni G., Azzini E., Comitato R., Sancak O., Barella L., Virgili F. (2014). Vitamin C supplementation modulates gene expression in peripheral blood mononuclear cells specifically upon an inflammatory stimulus: A pilot study in healthy subjects. Genes Nutr..

[242] Dawson W., West G.B. (1965). The influence of ascorbic acid on histamine metabolism in guinea-pigs. Br. J. Pharmacol. Chemother..

[243] Nandi B.K., Subramanian N., Majumder A.K., Chatterjee I.B. (1974). Effect of ascorbic acid on detoxification of histamine under stress conditions. Biochem. Pharmacol..

[244] Subramanian N., Nandi B.K., Majumder A.K., Chatterjee I.B. (1973). Role of L-ascorbic acid on detoxification of histamine. Biochem. Pharmacol..

[245] Chatterjee I.B., Gupta S.D., Majumder A.K., Nandi B.K., Subramanian N. (1975). Effect of ascorbic acid on histamine metabolism in scorbutic guinea-pigs. J. Physiol..

[246] Clemetson C.A. (1980). Histamine and ascorbic acid in human blood. J. Nutr..

[247] Phokela S.S., Peleg S., Moya F.R., Alcorn J.L. (2005). Regulation of human pulmonary surfactant protein gene expression by 1alpha,25-dihydroxyvitamin D3. Am. J. Physiol. Lung Cell Mol. Physiol..

[248] Ovesen L., Brot C., Jakobsen J. (2003). Food contents and biological activity of 25-hydroxyvitamin D: A vitamin D metabolite to be reckoned with?. Ann. Nutr. Metab..

[249] Traber M.G., Vitamin E., Shils M.E., Shike M., Ross A.C., Caballero B., Cousins R. (2006). Modern Nutrition in Health and Disease.

[250] Mihajlovic M., Fedecostante M., Oost M.J., Steenhuis S.K.P., Lentjes E., Maitimu-Smeele I., Janssen M.J., Hilbrands L.B., Masereeuw R. (2017). Role of Vitamin D in Maintaining Renal Epithelial Barrier Function in Uremic Conditions. Int. J. Mol. Sci..

[251] Suzuki H., Kunisawa J. (2015). Vitamin-mediated immune regulation in the development of inflammatory diseases. Endocr. Metab. Immune Disord Drug Targets..

[252] Yin Z., Pintea V., Lin Y., Hammock B.D., Watsky M.A. (2011). Vitamin D enhances corneal epithelial barrier function. Investig. Ophthalmol. Vis. Sci..

[253] Wu D., Meydani S.N. (2017). Vitamin E, immunity, and infection. Nutrition, Immunity, and Infection.

[254] Chavance M., Herbeth B., Fournier C., Janot C., Venhes G. (1989). Vitamin status, immunity and infections in an elderly population. Eur. J. Clin. Nutr..

[255] Wu D., Meydani S.N. (2014). Age-associated changes in immune function: Impact of vitamin E intervention and the underlying mechanisms. Endocr. Metab. Immune. Disord. Drug Targets.

[256] Panche A.N., Diwan A.D., Chandra S.R. (2016). Flavonoids: An overview. J. Nutr. Sci..

[257] Ravishankar D., Rajora A.K., Greco F., Osborn H.M. (2013). Flavonoids as prospective compounds for anti-cancer therapy. Int. J. Biochem. Cell Biol..

[258] Hodek P., Trefil P., Stiborová M. (2002). Flavonoids-potent and versatile biologically active compounds interacting with cytochromes P450. Chem. Biol. Interact..

[259] Liu Y., Jing Y.Y., Zeng C.Y., Li C.G., Xu L.H., Yan L., Bai W.-J., Zha Q.-B., Ouyang D.-Y., He X.-H. (2018). Scutellarin suppresses NLRP3 inflammasome activation in macrophages and protects mice against bacterial sepsis. Front Pharmacol..

[260] Dini I., Falanga D., Di Lorenzo R., Tito A., Carotenuto G., Zappelli C., Grumetto L., Sacchi A., Laneri S., Apone F. (2021). An Extract from *Ficus carica* Cell Cultures Works as an Anti-Stress Ingredient for the Skin. Antioxidants.

[261] Kopustinskiene D.M., Jakstas V., Savickas A., Bernatoniene J. (2020). Flavonoids as Anticancer Agents. Nutrients.

[262] Gupta S.C., Kunnumakkara A.B., Aggarwal S., Aggarwal B.B. (2018). Inflammation, a Double-Edge Sword for Cancer and Other Age-Related Diseases. Front. Immunol..

[263] Sargiacomo C., Sotgia F., Lisanti M.P. (2020). COVID-19 and chronological aging: Senolytics and other anti-aging drugs for the treatment or prevention of corona virus infection?. Aging.

[264] Smith M.S., Smith J.C. (2020). Repurposing therapeutics for COVID-19: Supercomputer-based docking to the SARS-CoV-2 Viral Spike Protein and Viral Spike Protein-Human ACE2 Interface. ChemRxiv..

[265] Ginwala R., Bhavsar R., Chigbu D.G.I., Jain P., Khan Z.K. (2019). Potential Role of Flavonoids in Treating Chronic Inflammatory Diseases with a Special Focus on the Anti-Inflammatory Activity of Apigenin. Antioxidants.

[266] Chirumbolo S. (2010). The role of quercetin, flavonols and flavones in modulating inflammatory cell function. Inflamm. Allergy Drug Targets.

[267] Verbeek R., Plomp A.C., van Tol E.A., van Noort J.M. (2004). The flavones luteolin and apigenin inhibit in vitro antigen-specific proliferation and interferon-gamma production by murine and human autoimmune T cells. Biochem. Pharmacol..

[268] Alschuler L., Weil A., Horwitz R., Stamets P., Chiasson A.M., Crocker R., Maizes V. (2020). Integrative considerations during the COVID-19 pandemic. Explore.

[269] Guo T.L., Mc Cay J.A., Zhang L.X., Brown R.D., You L., Karrow N.A. (2001). Genistein modulations immune responses and increases host resistance to B16F10 tumor in adult female B6cF1 mice. J Nutr..

[270] Sakai T., Kogiso M. (2008). Soy isoflavones and immunity. J. Investig. Med..

[271] Masilamani M., Wei J., Bhatt S., Paul M., Yakir S., Sampson H.A. (2011). Soybean isoflavones regulate dendritic cell function and suppress allergic sensitization to peanut. J. Allergy Clin. Immunol..

[272] Wei J., Bhatt S., Chang L.M., Sampson H.A., Masilamani M. (2012). Isoflavones, genistein and daidzein, regulate mucosal immune response by suppressing dendritic cell function. PLoS ONE..

[273] Smith B.N., Dilger R.N. (2018). Immunomodulatory potential of dietary soybean-derived isoflavones and saponins in pigs. J. Anim. Sci..

[274] Jin X., Wang S., Zhao X., Jin Q., Fan C., Li J., Shan Z., Teng W. (2016). Coumestrol inhibits autoantibody production through modulating Th1 response in experimental autoimmune thyroiditis. Oncotarget.

[275] Sirotkin A.V., Harrath A.H. (2014). Phytoestrogens and their effects. Eur. J. Pharmacol..

[276] Park J., Kim S.H., Cho D., Kim T.S. (2005). Formononetin, a phyto-oestrogen, its metabolites up-regulate interleukin-4 production in activated T cells via increased AP-1 DNA binding activity. Immunology.

[277] Kojima H., Takeda Y., Muromoto R., Takahashi M., Hirao T., Takeuchi S., Jetten A.M., Matzuda T. (2015). Isoflavones enhance interleukin-17 gene expression via retinoic acid receptor-related orphan receptors alpha and gamma. Toxicology.

[278] Abron J.D., Singh N.P., Price R.L., Nagarkatti M., Nagarkatti P.S., Singh U.P. (2018). Genistein induces macrophage polarization and systemic cytokine to ameliorate experimental colitis. PLoS ONE.

[279] Mace T.A., Ware M.B., King S.A., Loftus S., Farren M.R., McMichael E., Scoville S., Geraghty C., Young G., Carson W.E. (2019). Soy isoflavones and their metabolites modulate cytokine-induced natural killer cell function. Sci. Rep..

[280] Dia V.P., Berhow M.A., Gonzalez De Mejia E. (2008). Bowman-Birk inhibitor and genistein among soy compounds that synergistically inhibit nitric oxide and prostaglandin E2 pathways in lipopolysaccharide-induced macrophages. J. Agric. Food Chem..

[281] Amalraj A., Pius A., Gopi S., Gopi S. (2017). Biological activities of curcuminoids, other biomolecules from turmeric and their derivatives—A review. J. Tradit. Complement. Med..

[282] Kohli K., Ali J., Ansari M.J., Raheman Z. (2005). Curcumin: A natural antiinflammatory agent. Indian J. Pharmacol..

[283] Maiti P., Dunbar G.L. (2018). Use of Curcumin, a Natural Polyphenol for Targeting Molecular Pathways in Treating Age-Related Neurodegenerative Diseases. Int. J. Mol. Sci..

[284] Wanninger S., Lorenz V., Subhan A., Edelmann F.T. (2015). Metal complexes of curcumin—Synthetic strategies, structures and medicinal applications. Chem. Soc. Rev..

[285] Itokawa H., Shi Q., Akiyama T., Morris-Natschke S.L., Lee K.-H. (2008). Recent advances in the investigation of curcuminoids. Chin. Med..

[286] Chan K.K., Bin Hamid M.S., Webster R.D. (2018). Quantification of capsaicinoids in chillies by solid-phase extraction coupled with voltammetry. Food Chem..

[287] Srinivasan K. (2016). Biological Activities of Red Pepper (Capsicum annuum) and Its Pungent Principle Capsaicin: A Review. Crit. Rev. Food Sci. Nutr..

[288] Joe B., Lokesh B.R. (1997). Effect of curcumin and capsaicin on arachidonic acid metabolism and lysosomal enzyme secretion by rat peritoneal macrophages. Lipids.

[289] Anandakumar P., Kamaraj S., Jagan S., Ramakrishnan G., Asokkumar S., Naveenkumar C., Raghunandhakumar S., Devaki T. (2012). Capsaicin inhibits benzo(a)pyrene-induced lung carcinogenesis in an in vivo mouse model. Inflamm. Res..

[290] Zhang L.L., Yan Liu D., Ma L.Q., Luo Z.D., Cao T.B., Zhong J., Yan Z.C., Wang L.J., Zhao Z.G., Zhu S.J. (2007). Activation of transient receptor potential vanilloid type-1 channel prevents adipogenesis and obesity. Circ. Res..

[291] Zhang S., Ma X., Zhang L., Sun H., Liu X. (2017). Capsaicin reduces blood glucose by increasing insulin levels and glycogen content better than capsiate in streptozotocin-induced diabetic rats. J. Agric. Food Chem..

[292] Saito M., Matsushita M., Yoneshiro T., Okamatsu-Ogura Y. (2020). Brown Adipose Tissue, Diet-Induced Thermogenesis, and Thermogenic Food Ingredients: From Mice to Men. Front. Endocrinol..

[293] Ramirez D.A., Locatelli D.A., González R.E., Cavagnaro P.F., Camargo A.B. (2017). Analytical methods for bioactive sulfur compounds in *Allium*: An integrated review and future directions. J. Food Comp. Anal..

[294] Pinto J.T., Krasnikov B.F., Cooper A.J.L. (2006). Redox-sensitive proteins are potential targets of garlic-derived mercaptocysteine derivatives. J. Nutr..

[295] Wang H.C., Pao J., Lin S.Y., Sheen L.Y. (2012). Molecular mechanisms of garlic-derived allyl sulfides in the inhibition of skin cancer progression. Ann. N. Y. Acad. Sci..

[296] Nuutila A.M., Puupponen-Pimiä R., Aarni M., Oksman-Caldentey K.-M. (2003). Comparison of antioxidant activities of onion and garlic extracts by inhibition of lipid peroxidation and radical scavenging activity. Food Chem..

[297] Das A., Banik N.L., Ray S.K. (2007). Garlic compounds generate reactive oxygen species leading to activation of stress kinases and cysteine proteases for apoptosis in human glioblastoma T98G and U87MG cells. Cancer.

[298] Burt S. (2004). Essential oils: Their antibacterial properties and potential applications in foods-a review. Int. J. Food Microbiol..

[299] Sivakumar P.M., Kumar T.M., Doble M. (2009). Antifungal activity, mechanism and QSAR studies on chalcones. Chem. Biol. Drug Des..

[300] Rozmer Z., Perjési P. (2016). Naturally occurring chalcones and their biological activities. Phytochem. Rev..

[301] Kurita N., Miyaji M., Kurane R., Takahara Y., Ichimura K. (1979). Antifungal activity and molecular orbital energies of aldehyde compounds from oils of higher plants. Agric. Biol. Chem..

[302] Babu K.S., Li X.C., Jacob M.R., Zhang Q., Khan S., Ferreia D., Clark A.M. (2006). Synthesis, antifungal activity, and structure-activity relationships of coruscanone A. analogs. J. Med. Chem..

[303] Monzote L., Stamberg W., Staniek K., Gille L. (2009). Toxic effects of carvacrol, caryophyllene oxide, and ascaridole from essential oil of Chenopodium ambrosioides on mitochondria. Toxicol. Appl. Pharmacol..

[304] Cao Y., Gu W., Zhang J., Chu Y., Ye X., Hu Y., Chen J. (2013). Effects of chitosan, aqueous extract of ginger, onion and garlic on quality and shelf life of stewed-pork during refrigerated storage. Food Chem..

[305] Souza V.G.L., Pires J.R.A., Vieira É.T., Coelhoso I.M., Duarte M.P., Fernando A.L. (2018). Shelf Life Assessment of Fresh Poultry Meat Packaged in Novel Bionanocomposite of Chitosan/Montmorillonite Incorporated with Ginger Essential Oil. Coatings.

[306] Khaledian Y., Pajohi-Alamoti M., Bazargani-Gilani B. (2019). Development ofcellulose nanofibers coating incorporated with ginger essential oiland citric acid to extend the shelf life of ready-to-cook barbecuechicken. J. Food Process. Preserv..

[307] Mansouri S., Pajohi-Alamoti M., Aghajani N., Bazargani-Gilani B., Nourian A. (2020). Stability and antibacterial activity of Thymus daenensis L. essential oil nanoemulsion in mayonnaise. J. Sci. Food Agric..

[308] Esmaeili H., Cheraghi N., Khanjari A., Rezaeigolestani M., Basti A.A., Kamkar A., Aghaee E.M. (2020). Incorporation of nanoencapsulated garlic essential oil into edible films: A novel approach for extending shelf life of vacuum-packed sausages. Meat Sci..

[309] Hernández-Ochoa L., Aguirre-Prieto Y.B., Nevárez-Moorillón G.V., Gutierrez-Mendez N., Salas-Munoz E. (2014). Use of essential oils and extracts from spices in meat protection. J. Food Sci. Technol..

[310] Salma U., Saha S.K., Sultana S., Ahmed S.M., Haque S.D., Mostaqim S. (2019). The Antibacterial Activity of Ethanolic Extract of Cinnamon (*Cinnamomum zeylanicum*) against two Food Borne Pathogens: *Staphylococcus aureus* And *Escherichia coli*. Mymensingh Med. J..

[311] Shan B., Cai Y.Z., Brooks J.D., Corke H. (2011). Potential application of spice and herb extracts as natural preservatives in cheese. J. Med. Food.

[312] Badea G., Bors A.G., Lacatusu I., Oprea O., Ungureanu C., Stan R., Meghea A. (2015). Influence of basil oil extract on the antioxidant and antifungal activities of nanostructured carriers loaded with nystatin. Comptes Rendus Chim..

[313] El-Soud N.H.A., Deabes M., . El-Kassem L.A., Khalil M. (2015). Chemical composition and antifungal activity of *Ocimum basilicum* L. essential oil. Maced. J. Med. Sci..

[314] Saggiorato A.G., Gaio I., Treichel H., de Oliveira D., Cichoski A.J., Cansian R.L. (2012). Antifungal activity of basil essential oil (Ocimum basilicum L.): Evaluation in vitro and on an Italian-type sausage surface. Food Bioproc. Technol..

[315] Kumar R., Ghoshal G., Goyal M. (2021). Effect of basil leaves extract on modified moth bean starch active film for eggplant surface coating. LWT. Food Sci.Technol..

[316] Gundewadi G., Sarkar D.J., Rudra S.G., Singh D. (2018). Preparation of basil oil nanoemulsion using Sapindus mukorossi pericarp extract: Physico-chemical properties and antifungal activity against food spoilage pathogens. Ind. Crop. Prod..

[317] Santos N.S.T., Aguiar A.J.A.A., de Oliveira C.E.V., de Sales C.V., Silva S.M., Silva R.S., Stamford T.C.M., de Souza E.L. (2012). Efficacy of the application of a coating composed of chitosan and Origanum vulgare L. essential oil to control *Rhizopus stolonifer* and *Aspergillus niger* in grapes (*Vitis labrusca* L.). Food Microbiol..

[318] Wang C.Y., Wang S.Y., Yin J.J., Parry J., Yu L.L. (2007). Enhancing Antioxidant, Antiproliferation, and free radical scavenging activities in strawberries with essential oils. J. Agric. Food Chem..

[319] Valero D., Valverde J.M., Martı´nez-Romero D., Guille´n F., Castillo S., Serrano M. (2006). The combination of modified atmosphere packaging with eugenol or thymol to maintain quality, safety and functional properties of table grapes. Postharvest Biol. Technol..

[320] Miao L., Walton W.C., Wang L., Li L., Wang Y. (2019). Characterization of polylactic acids-polyhydroxybuty rate based packaging film with fennel oil, and its application on oysters. Food Packag. Shelf Life..

[321] Wang C.Y., Wang S.Y., Chen C. (2008). Increasing antioxidant activity and reducing decay of blueberries by essential oils. J. Agric. Food. Chem..

[322] Jin P., Wang S.Y., Gao H., Chen H., Zheng Y., Wang C.Y. (2012). Effect of cultural system and essential oil treatment on antioxidant capacity in raspberries. Food Chem..

[323] Kato H.C.A., Peixoto Joele M.R.S., Sousa C.L., Ribeiro S.C.A., Lourenço L.F.H. (2017). Evaluation of the shelf life of tambaqui fillet processed by the sous vide method. J. Aquat. Food Prod. Technol..

[324] Toyohara M., Murata M., Ando M., Kubota S., Sakaguchi M., Toyohara H. (1999). Texture changes associated with insolubilization of sarcoplasmic proteins during salt-vinegar curing of fish. Food Sci. J..

[325] Stelzleni A.M., Ponrajan A., Harrison M.A. (2013). Effects of buffered vinegar and sodium dodecyl sulfate plus levulinic acid on Salmonella Typhimurium survival, shelf-life, and sensory characteristics of ground beef patties. Meat Sci..

[326] Shi C., Cui J., Na Q., Luo Y., Han L., Hang W. (2017). Effect of ginger extract and vinegar on atp metabolites, imp-related enzyme activity, reducing sugars and phosphorylated sugars in silver carp during postslaughter storage. Int. J. Food Sci. Technol..

[327] Uvere P.O., Onyekwere E.U., Ngoddy P.O. (2010). Production of maize-bambara groundnut complementary foods fortified pre-fermentation with processed foods rich in calcium, iron, zinc and provitamin A. J. Sci. Food Agric..

[328] Bakrm S.A., Salihin B.A. (2013). Effects of inclusion of Allium sativum and Cinnamomumverum in milk on the growth and activity of lactic acid bacteria during yoghurt fermentation Amer-Euras. J. Agric. Environ. Sci..

[329] Helal A., Tagliazucchi D. (2018). Impact of in-vitro gastro-pancreatic digestion on polyphenols and cinnamaldehyde bioaccessibility and antioxidant activity in stirred cinnamon-fortified yoghurt. LWT Food Sci. Technol..

[330] Srivastava P., Prasad S.G.M., Mohd N.A., Prasad M. (2015). Analysis of antioxidant activity of herbal yoghurt prepared from different milk. J. Pharma. Innov..

[331] Amirdivani S., Baba A.S. (2011). Changes in yoghurt fermentation characteristics, and antioxidant potential and in vitro inhibition of angiotensin-1 converting enzyme upon the inclusion of peppermint, dill and basil. LWT Food Sci. Technol..

[332] Carocho M., Barros L., Barreira J.C., Calhelha R.C., Sokovi´c M., Fernández-Ruiz V., Buelga C.S., Morales P., Ferreira I.C. (2016). Basil as functional and preserving ingredient in “serra da estrela” cheese. Food Chem..

[333] Sikora M., Złotek U., Kordowska-Wiater M., Świeca M. (2020). Effect of Basil Leaves and Wheat Bran Water Extracts on Antioxidant Capacity, Sensory Properties and Microbiological Quality of Shredded Iceberg Lettuce during Storage. Antioxidants.

[334] Josipović R., Kneževi Z.M., Frece J., Markov K., Kazazić S., Mrvčić J. (2015). Improved properties and microbiological safety of novel cottage cheese containing spices. Food Technol. Biotechnol..

[335] Najgebauer L.D., Grega T., Sady M. (2009). The quality and storage stability of butter made from sour cream with addition of dried sage and rosemary. Biotechnol. Anim. Husb..

[336] Farag R.S., Ali M.N., Taga S.H. (1990). Use of some essential oils as natural preservatives for butter. J. Am. Oil Chem. Soc..

[337] Pinto S.V., Patel A.M., Jana A.H. (2009). Evaluation of different forms of ginger as flavouring in herbal ice cream. Int. J. Food Sci. Technol. Nutr..

[338] Ham Y.-K., Hwang K.-E., Song D.-H., Choi J.-H., Choi Y.-S., Kim H.-W. (2019). Relationship between the antioxidant capacity of soy sauces and its impact on lipid oxidation of beef patties. Meat Sci..

[339] Honda M., Kageyama H., Hibino T., Takemura R., Goto M., Fukaya T. (2019). Enhanced Z-isomerization of tomato lycopene through the optimal combination of food ingredients. Sci Rep..

[340] González-Hedström D., Granado M., Inarejos-García A.M. (2020). Protective effects of extra virgin olive oil against storage-induced omega 3 fatty acid oxidation of algae oil. NFS J..

[341] Zhang Y., Wang Y., Zhang F., Wang K., Liu G., Yang M., Zhang D. (2015). Allyl methyl disulfide inhibits IL-8 and IP-10 secretion in intestinal epithelial cells via the NF-kappa B signaling pathway. Inter. Immunopharmac..

[342] Bordoloi R., Ahmed N.S., Dora K.C., Chowdhury S. (2017). Effect of cooking on antioxidant and antimicrobial property of spices. Biochem. Cell. Arch..

[343] Neves T.M., da Cunha D.T., de Rosso V.V., Domene S.M.Á. (2021). Effects of seasoning on the formation of heterocyclic amines and polycyclic aromatic hydrocarbons in meats: A meta-analysis. Compr. Rev. Food Sci. Food Saf..

[344] IARC (2015). Red Meat and Processed Meat.

[345] Shabbir A.M., Raza A., Faqir M.A., Khan M.R., Suleria H.A.R. (2015). Effect of thermal treatment on meat proteins with special reference to heterocyclic aromatic amines (HAAs). Crit. Rev. Food Sci. Nutr..

[346] Janoszka B., Nowak A., Szumska M., Śnieżek E., Tyrpień-Golder K. (2019). Human exposure to biologically active heterocyclic aromatic amines arising from thermal processing of protein rich food. Wiadomosci Lek..

[347] Liang T.F., Wei F.F., Lu Y., Kodani Y., Nakada M., Miyakawa T., Tanokura M. (2015). Comprehensive nmr analysis of compositional changes of black garlic during thermal processing. J. Agric. Food Chem..

[348] Mashilipa C., Wang Q., Slevin M., Ahmed N. (2011). Antiglycation and antioxidant properties of soy sauces. Med. Food J..

[349] Santos C.S.P., Cruz R., Cunha S.C., Casal S. (2013). Effect of cooking on olive oil quality attributes. Food Res. Int..

[350] Anh N.H., Kim S.J., Long N.P., Min J.E., Yoon Y.C., Lee E.G., Kim M., Kim T.J., Yang Y.Y., Son E.Y. (2020). Ginger on Human Health: A Comprehensive Systematic Review of 109 Randomized Controlled Trials. Nutrients.

[351] Sellami M., Slimeni O., Pokrywka A., Kuvačić G., Hayes L.D., Milic M., Padulo J. (2018). Herbal medicine for sports: A review. J. Int. Soc. Sports Nutr..

[352] Onuora C., Ofili C.T., Salawu S., Elimian I., Shehu H. (2019). Therapeutic Effects of Garlic: A Review. Sci. J. Biol. Life Sci..

[353] Ouzir M., El Bairi K., Amzazi S. (2016). Toxicological properties of fenugreek (Trigonella foenum graecum). Food Chem. Toxicol..

[354] Liddle M., Hull C., Liu C., Powell D. (2006). Contact urticaria from curcumin. Dermatitis.

[355] Lopez-Lazaro M. (2008). Anticancer and carcinogenic properties of curcumin: Considerations for its clinical development as a cancer chemopreventive and chemotherapeutic agent. Mol. Nutr. Food Res..

